# Survival of Zirconium-Based Metal–Organic Framework Crystallinity at Extreme Pressures

**DOI:** 10.1021/acs.inorgchem.2c04428

**Published:** 2023-06-16

**Authors:** Georgina P. Robertson, Sara Mosca, Celia Castillo-Blas, Florencia A. Son, Omar K. Farha, David A. Keen, Simone Anzellini, Thomas D. Bennett

**Affiliations:** † Department of Materials Science and Metallurgy, University of Cambridge, 27 Charles Babbage Road, Cambridge, Cambridgeshire CB3 0FS, U.K.; ‡ Diamond Light Source Ltd., Diamond House, Harwell Science and Innovation Campus, Didcot, Oxfordshire OX11 0DE, U.K.; § Central Laser Facility, STFC, Rutherford Appleton Laboratory, Harwell Science and Innovation Campus, Didcot, Oxfordshire OX11 0QX, U.K.; ∥ Department of Chemistry and International Institute of Nanotechnology, Northwestern University, 2145 Sheridan Road, Evanston, Illinois 60208, United States; ⊥ ISIS Facility, Rutherford Appleton Laboratory, Harwell Science and Innovation Campus, Didcot, Oxfordshire OX11 0QX, U.K.

## Abstract

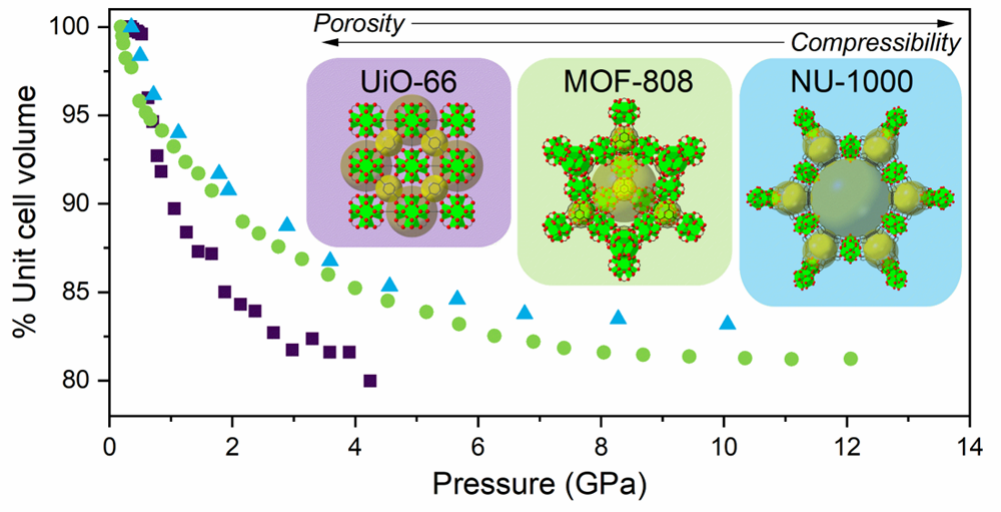

Recent research on metal–organic frameworks (MOFs) has shown a shift from considering only the crystalline high-porosity phases to exploring their amorphous counterparts. Applying pressure to a crystalline MOF is a common method of amorphization, as MOFs contain large void spaces that can collapse, reducing the accessible surface area. This can be either a desired change or indeed an unwanted side effect of the application of pressure. In either case, understanding the MOF’s pressure response is extremely important. Three such MOFs with varying pore sizes (UiO-66, MOF-808, and NU-1000) were investigated using *in situ* high-pressure X-ray diffraction and Raman spectroscopy. Partial crystallinity was observed in all three MOFs above 10 GPa, along with some recovery of crystallinity on return to ambient conditions if the frameworks were not compressed above thresholds of 13.3, 14.2, and 12.3 GPa for UiO-66, MOF-808, and NU-1000, respectively. This threshold was marked by an unexpected increase in one or more lattice parameters with pressure in all MOFs. Comparison of compressibility between MOFs suggests penetration of the pressure-transmitting oil into MOF-808 and NU-1000. The survival of some crystallinity above 10 GPa in all of these MOFs despite their differing pore sizes and extents of oil penetration demonstrates the importance of high-pressure characterization of known structures.

## Abstract 2

Zirconium-based metal−organic frameworks UiO-66, MOF-808, and NU-1000 were investigated *in situ* under extreme pressures using X-ray diffraction. They all showed surviving crystallinity at pressures of >10 GPa. Their opposing trends of compressibility and porosity are ascribed to varying interactions with the silicone oil pressure-transmitting medium.

## Introduction

Metal–organic frameworks (MOFs) are hybrid materials formed of inorganic nodes called secondary building units (SBUs), connected by organic molecules acting as linkers. Their high porosity and customizable chemical and physical properties through different SBU/linker combinations mean they have many potential applications, including gas sensing and separation, medical imaging and therapeutics, and catalysis.^1^

These frameworks have a variety of responses to pressure. Flexible MOFs can undergo reversible “breathing” transitions to drastically alter their unit cell volume, and even so-called “rigid” MOFs can experience linker rotation under pressure.^2,3^ Alternatively, the nanoporous architectures of MOFs may collapse under pressure due to the presence of void spaces. In general, MOFs have bulk moduli that are much lower than those of similarly structured inorganic equivalents, most clearly seen when comparing zeolites with zeolitic imidazolate frameworks (ZIFs).^4^ MOFs can also undergo shear softening that further decreases their bulk moduli during compression.^5^ Loss of crystallinity is therefore commonly observed in the relatively few MOFs to have been experimentally measured under pressure.^6−12^ This process is termed pressure-induced amorphization (PIA). While amorphization can be beneficial in some contexts,^13,14^ collapse of the pores causes a loss of surface area that is detrimental to most applications.^15^ Understanding the mechanism of amorphization is therefore of utmost importance, whether in seeking to prevent unwanted collapse or indeed to introduce a controlled loss of crystallinity.

PIA at low pressures is common, with MOF-5, ZIF-8, and Zn_2_bdc_2_dabco amorphizing at ∼0.3 GPa^6−8^ and ZIF-4 and ZIF-62 amorphizing at ∼5 GPa.^9,10^ However, it has recently been demonstrated that MOFs can survive to much higher pressures when large molecules such as oils are inside the pores, preventing their collapse. For example, PIA in MIL-101 shifts from 0.4 to 13 GPa when a nonpenetrating solid NaCl pressure-transmitting medium (PTM) is replaced with penetrating polydimethylsiloxane (PDMS) silicone oil.^11^ While the solid PTM’s low hydrostatic limit may play a role in promoting PIA, a standard deviation in pressure of <0.1 GPa was maintained to minimize the effect of shear stresses, allowing comparison with the 12 GPa limit of the fluids. Similarly, MOF-5 survives to 9 GPa with Daphne oil as a penetrating PTM, whereas it amorphizes at 3.24 GPa with the penetrating small molecule diethyl formamide.^8,12^ This also demonstrates that the penetration of small molecule PTMs does not always stabilize a framework against pressure, as the molecules can be forced out again during compression, where larger molecules may be trapped within the pores. Interactions of PTM with MOFs are complex, and individual cases should be investigated experimentally before any assumptions can be made about their effect on a framework’s mechanical properties.

Zirconium-based MOFs with an octahedral Zr_6_O_8_ SBU make up a well-known family and are investigated for many applications, including catalysis, drug delivery, and adsorption of toxic compounds.^16,17^ Despite their promising properties for these uses, and the prototypical member UiO-66’s documented stability under pressure,^18^ to the best of our knowledge, this family has not been studied under pressures of >5 GPa.^19^ As such, PIA has not been observed in UiO-66, and the mechanical behavior of semicrystalline or amorphous phases of it or other similar MOFs has not been characterized.

**Figure 1:**
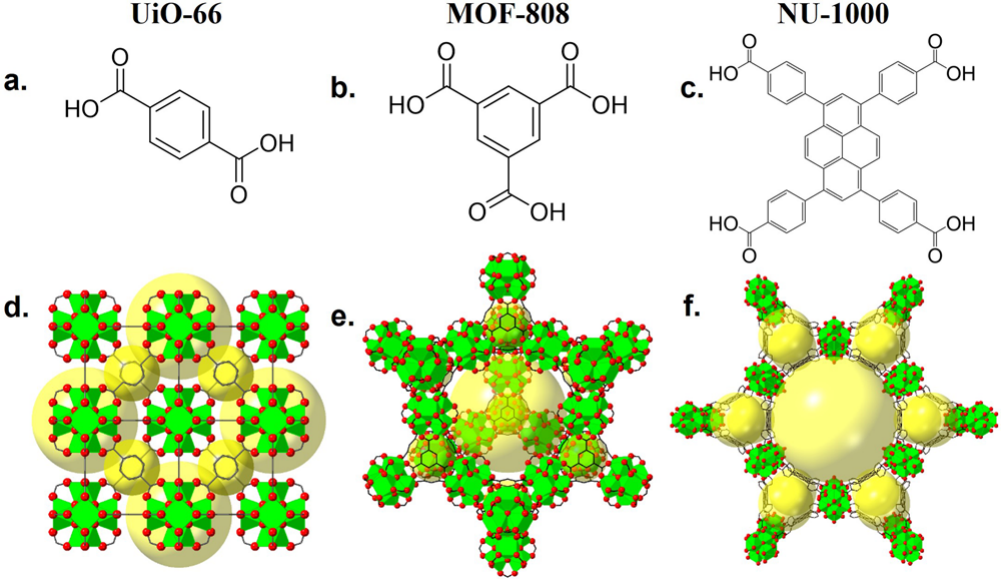
(a–c) Linkers and (d–f) structures of the Zr_6_O_8_ node-based MOFs (a and d) UiO-66, (b and e) MOF-808, and (c and f) NU-1000. Zr nodes are colored green, oxygens red, and carbons black. Hydrogens have been omitted for the sake of clarity, and cavities illustrating pore structure are colored yellow.

To address this, we here present a study in which three Zr-MOFs were compressed with silicone oil AP100 to investigate its effect on their mechanical behavior and explore the oil’s potential to stabilize their crystalline phases against high pressure. UiO-66 was chosen as it is the most well documented zirconium-based MOF. MOF-808 and NU-1000 were chosen as they share the same inorganic cluster as UiO-66 but differ in ligand and structure, giving a range of pore dimensions for comparison (Table 1 and Figure 1). The SBU is formed by an octahedron of Zr(IV) ions with μ_3_-OH or μ_3_-O moieties coordinated at each face. UiO-66 has 12 ditopic linkers connected to each metal cluster, which forms a cubic structure in space group *Fm*3̅*m*. MOF-808 is also cubic, with space group *Fd*3̅*m*, and with each SBU connected to six tritopic linkers. Finally, NU-1000 forms a hexagonal structure in space group *P*6/*mmm*, with metal clusters coordinated to eight tetratopic linkers.

**Table 1:** Chemical Formulae and Structural Details for the Three Zirconium-Based MOFs

MOF	linker	theoretical formula	topology	connectivity
UiO-66	1,4-benzenedicarboxylic acid (H_2_BDC)	Zr_6_O_4_(OH)_4_(C_8_H_4_O_4_)_6_	*fcu*	12
MOF-808	1,3,5-benzenetricarboxylic acid (H_3_BTC)	Zr_6_O_4_(OH)_4_(C_9_H_3_O_6_)_2_(HCOO)_6_	*spn*	6
NU-1000	1,3,6,8-tetrakis(*p*-benzoate)pyrene (H_4_TBAPy)	Zr_6_O_4_(OH)_8_(OH_2_)_4_(C_44_H_22_O_8_)_2_	*csq*	8

## Methods

Polycrystalline MOF samples were synthesized using methods from the literature.^20−23^ Heating under dynamic vacuum (activation) was performed upon synthesis, and before each experiment, to ensure removal of solvent from within the pores. The resultant powders were characterized with powder X-ray diffraction (PXRD), gas adsorption measurements, elemental analysis, nuclear magnetic resonance (NMR), thermal analysis, and Fourier transform infrared (FTIR) spectroscopy.

### Ambient-Pressure PXRD

Powders of the selected MOFs were loaded into a 5 mm diameter inset in a silicon substrate, which was rotated during collection of data from 2θ = 1.5° to 50°. A Bruker D8 Advance diffractometer equipped with a position-sensitive LynxEye detector was used in Bragg–Brentano geometry. Cu Kα radiation (λ = 1.5418 Å) was used, with a variable divergence slit to prevent overillumination of the sample. Data were then corrected to appear as if taken by a fixed divergence slit illuminating a constant volume using the DIFFRAC.EVA software.^24^ Raw data were converted to .xy format using the PowDLL software suite^25^ and refined using the TOPAS-7 software suite.^26^

### *In Situ* High-Pressure PXRD

Membrane diamond anvil cells (DACs) equipped with diamonds with a culet diameter of 400 μm were used for the high-pressure PXRD experiment. Stainless steel foils (initial thickness of 150 μm) were used as gasket materials. The high-pressure chambers were created by preindenting the foils to a thickness of 40 μm and drilling 300 μm diameter holes in them using a spark-erosion machine. The powder samples were loaded under air into the DAC to completely fill the high-pressure chamber, and a grain of W was added as an X-ray gauge. Silicone oil AP100 (polyphenyl-methylsiloxane, hydrostatic limit of 7 GPa^27^) was used as a pressure-transmitting medium. Measurements were taken on beamline I15 at Diamond Light Source, UK, using a beam energy of 29.287 keV (wavelength of 0.4223 Å), and collected on a CdTe Pilatus 2M detector using a 90 s exposure time and rocking (1° for UiO-66 and 5° for MOF-808 and NU-1000).^28^ The detector geometry was calibrated with a LaB_6_ standard using the powder calibration routine of the DIOPTAS software suite.^29^ Masks were applied to the raw diffraction images on a per-image basis before they were azimuthally integrated in DIOPTAS. Lattice parameters were extracted from the obtained diffraction data through a Pawley refinement using the TOPAS-7 software suite. Literature values were used as a starting point for the refinement of each MOF. Bragg peak parameters were extracted using the Fityk software suite for analysis of the peak position, shape, and intensity to investigate loss of crystallinity.^30^ The pressure inside the high-pressure chamber was measured from the obtained volumetric compression of the tungsten gauge, following the equation of state calibration of Dorogokupets and Oganov.^31^ The bulk modulus for each MOF was calculated by fitting to Birch–Murnaghan equations of state with EoSfit7 GUI software.^32^

### High-Pressure Raman Spectroscopy

*In situ* Raman spectra were recorded using an inVia Renishaw Raman microscope.^33^ A 5× objective lens and an 830 nm excitation wavelength were used with a laser power of 5.5 mW and an exposure time of 15 s. Raman spectra were recorded in the spectral range of 150–1700 cm^–1^ (spectral resolution of 4 cm^–1^). A DAC equipped with diamonds with a culet diameter of 700 μm was used with a stainless steel gasket preindented to a thickness of 60 μm and with a 300 μm diameter hole. The gasket was prepared following the procedure described in the previous paragraph. As for the PXRD experiment, for each experimental pressure ramp, the high-pressure chamber of the DAC was completely filled with the powdered sample. A ruby chip was loaded with the sample and used as a pressure gauge. Silicone oil AP100 was used as a pressure-transmitting medium, and pressure was applied by manually tightening the DAC’s screws. The pressure inside the DAC was measured using the ruby fluorescence method, following the calibration of Dorogokupets and Oganov.^31^ Raman spectra of silicone oil AP100 were recorded under ambient conditions on a glass slide using the same microscope settings.

## Results

### Initial Characterization

The ambient-pressure MOF PXRD patterns were in good agreement with the simulated patterns of the expected crystal structures (Figure S1 and Table S1).^34,35^ Thermogravimetric analysis (TGA) (Figure S6 and Table S7), ^1^H NMR spectroscopy (Figures S3–S5), and elemental analysis (Table S3) were used to determine the nature and amount of the species coordinated at nonlinker positions in the three MOFs. These findings are detailed in the Supporting Information and summarized in Table 2. FTIR spectroscopy showed similar chemical bonding in each MOF as expected given their similar SBUs and linkers (Figure S7). Their Brunauer–Emmett–Teller (BET) areas and pore size distributions compared well to those in the literature (Table S2 and Figure S2).

**Table 2:** Defect Characterization Summary, Giving the Number of Linkers per SBU and the Calculated Formula of Each MOF from the Combined Techniques (DMF = *N,N*-dimethylformamide)

MOF	no. of linkers per SBU (theoretical)	no. of linkers per SBU (actual)	calculated formulas
UiO-66	6	5.6	Zr_6_O_4_(OH)_4_(OH_2_)_0.8_(C_8_H_4_O_4_)_5.6_Cl_0.8_·DMF
MOF-808	2	1.9	Zr_6_O_4_(OH)_8_(C_9_H_3_O_6_)_2_(HCOO)_1.44_Cl_0.56_(DMF)_0.56_
NU-1000	2	2.1	Zr_6_O_4_(OH)_7_(OH_2_)_4_(C_44_H_22_O_8_)_2_Cl

### High-Pressure Evolution

The structural evolution of the three Zr-based MOFs was investigated at ambient temperature and under quasi-hydrostatic conditions. True hydrostatic conditions could not be reached above 7 GPa due to the hydrostatic limit of the PTM.^27^ Data collected prior to compression from samples within the DACs agreed well with those collected *ex situ* on as-synthesized samples. Synchrotron XRD data were collected for all samples from near-ambient conditions, and then under compression, at pressure intervals varying between 0.5 and 4 GPa. With an increasing pressure on the UiO-66 sample, there was the expected shift of the peaks to larger angles due to the compression of the lattice, together with Bragg peak broadening and a reduction in intensity, until the peak shape and position changed negligibly with a further increase in pressure. Here, compression was halted at 4.25 GPa and then released to check the reversibility of the process (Figure S8). A second compression was then carried out using the same sample immediately after the return to ambient conditions. When 4.25 GPa was exceeded, an interesting shift of the most intense Bragg peak to smaller angles occurred. This peak is the 111 reflection, assuming no phase change had occurred (Figure S1). After this peak shift was observed, compression was again halted and released to check the reversibility of the process. The maximum pressure of the second ramp was 14.7 GPa.

**Figure 2:**
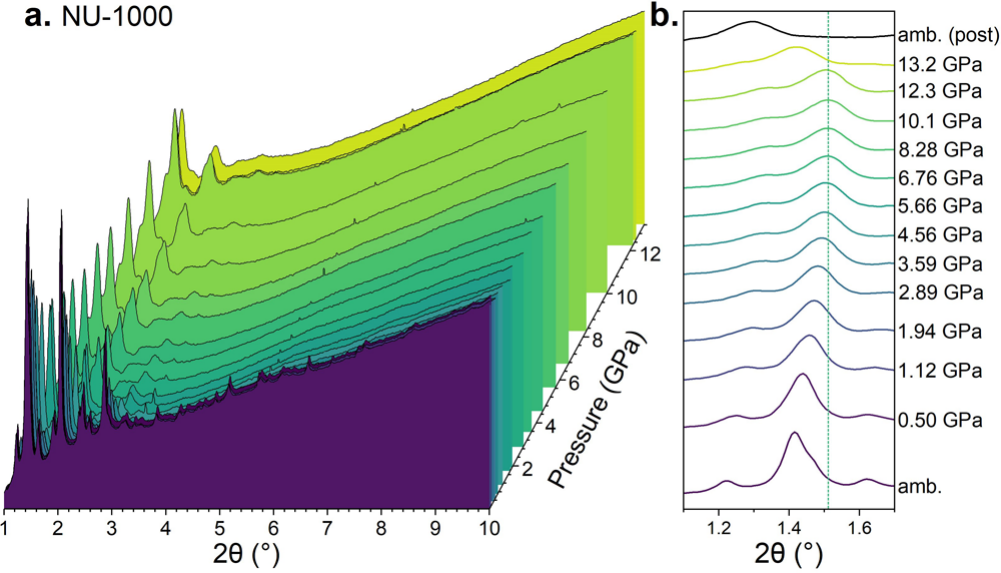
(a) XRD data of NU-1000 under increasing pressures. (b) The same data cropped to show the highest-intensity Bragg peak. The black trace at the top shows data under ambient conditions after compression. The vertical dotted line indicates the highest angle position of the peak. The high background is due to the diffuse scattering contribution from the DAC and other surrounding equipment.

The same procedure was carried out for MOF-808, with an initial compression maximum of 12.1 GPa and a second compression maximum of 19.3 GPa. A shift of the most intense Bragg peak [again the 111 reflection (Figure S1)] was again observed with the higher-pressure compression. For NU-1000, this peak shift to smaller angles [of the 22̅0 reflection (Figure S1)] was seen in the first compression to a maximum of 13.2 GPa due to an overshoot in the DAC membrane. A fresh sample was therefore reloaded for a compression to a lower maximum of 7.71 GPa, a pressure below which the peak shift to smaller angles was seen. Pawley refinements of all data for each MOF were possible using the initial crystalline structures (Tables S8–S10), although the broadness of features at the highest pressures causes some uncertainty in terms of the phase assignment. Figure 2 shows an example of the pressure-induced PXRD pattern evolution observed for NU-1000, where the first strong Bragg peak can be seen to move to a smaller angle after 12.3 GPa.

**Figure 3:**
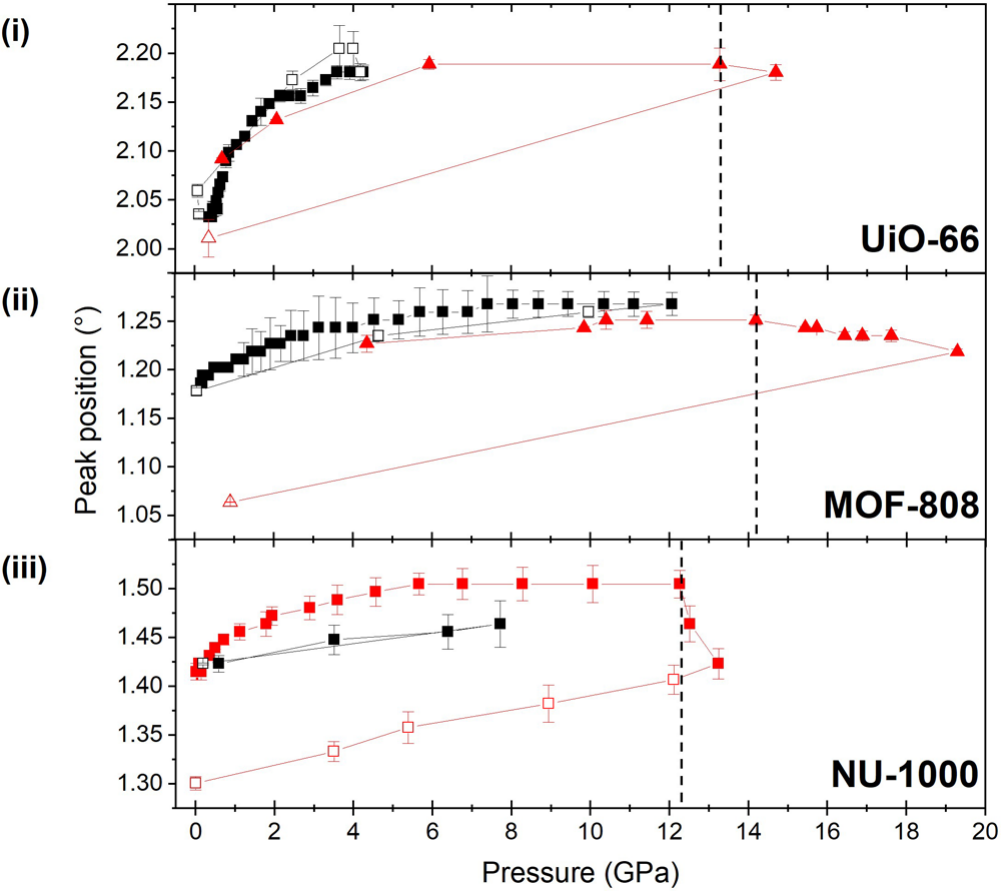
Pressure-induced shift of the position of the most intense Bragg peak of UiO-66, NU-1000, and MOF-808. Each MOF was compressed twice, to a lower (black) and higher (red) maximum. Filled and empty data points show compression and decompression ramps, respectively. Square data points indicate a sample starting from fully crystalline, and triangular data points indicate a sample that has undergone previous compression to a lower maximum. The pressure threshold before the peak shifts to smaller angles is shown with a dotted line. Data was taken with a wavelength of 0.4223 Å.

The evolution of the most intense Bragg peak’s position as a function of pressure was analyzed by peak fitting for all MOFs (Figure 3). The shift of this peak to smaller angles with an increase in pressure was seen above thresholds of 13.3, 14.2, and 12.3 GPa for UiO-66, MOF-808, and NU-1000, respectively. Although this shift was observed in all of the MOFs, the relative amount was different, with the maximum values observed in NU-1000 and the minimum values observed in UiO-66. The lattice parameters of each MOF at high pressures cannot be derived with high certainty, given the broad features of the diffraction pattern. Although the most intense features of the pattern of each MOF remain in similar positions, it is not possible to be sure if the original symmetry remains at extreme pressures or if a transition to an alternate crystalline phase might have taken place. 2θ positions are therefore displayed rather than *d* spacings, to avoid an implication that each peak unambiguously contains only one Bragg position at the highest pressures.

**Figure 4:**
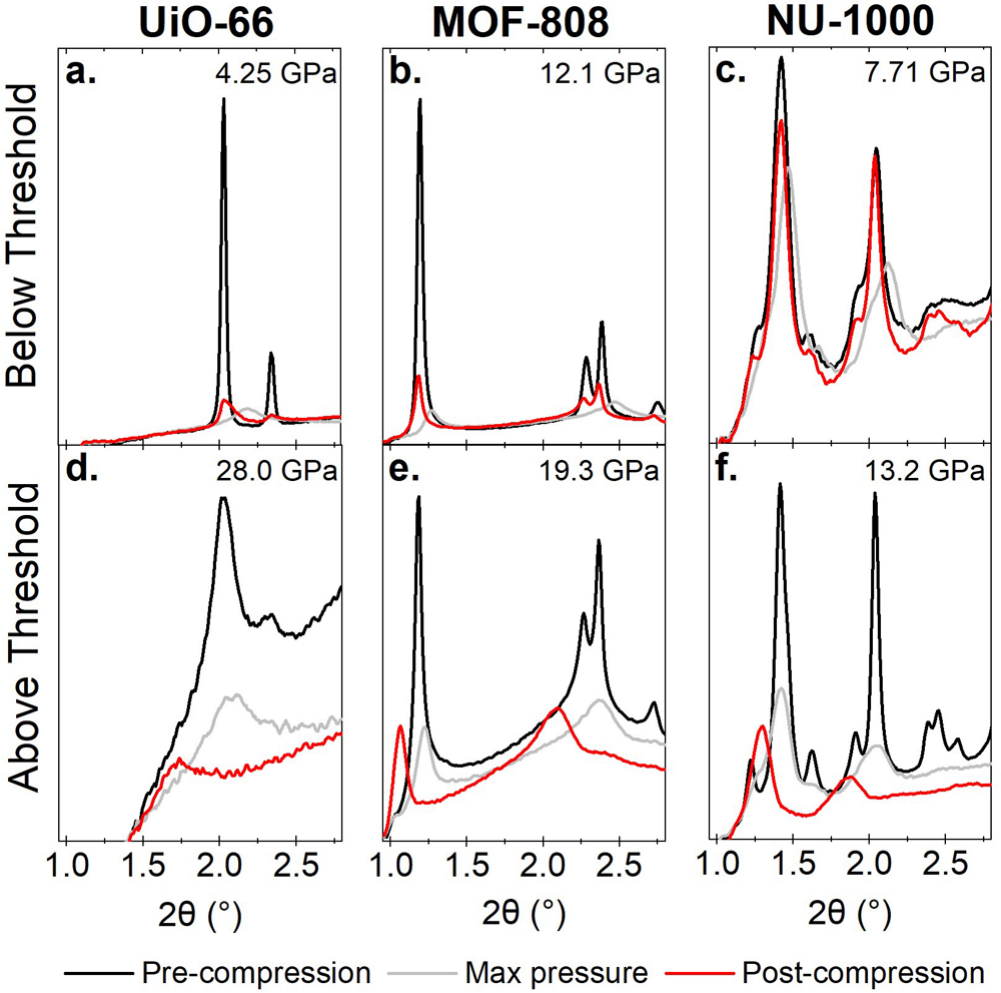
Comparison of low-angle XRD data before compression, at maximum pressure for the given ramp, and after decompression for (a and d) UiO-66, (b and e) MOF-808, and (c and f) NU-1000. Panels a–c show the data for each MOF when ramped to a lower maximum pressure. Panels d–f show the data for each MOF when ramped to a higher maximum pressure where the peak shift to smaller angles was observed. The maximum pressure for each ramp is given at the top right of each plot.

After a return to ambient conditions from the first compression ramp (below the threshold), the Bragg peaks in the PXRD patterns of each MOF recovered some peak shape and intensity. This shows some recovery of crystallinity (Figure 4a–c): the recovery is more pronounced in NU-1000 and MOF-808. However, after decompression from the higher maximum pressure of the second ramp (above the threshold), the Bragg features did not sharpen or increase in intensity (Figure 4d–f). This suggests no recovery of crystallinity upon decompression from this higher limit. Furthermore, there was a relative shift to a smaller angle of the main peaks upon decompression to ambient conditions, confirming an irreversible structural change after the second pressure ramp. This was not seen after the first ramp, which is shown by the post-decompression data in Figure 3.

Despite the loss of crystallinity, low-intensity but distinct Bragg features were still present for all MOFs after the second compression. An amorphous sample would be expected to have no distinguishable features in its XRD pattern apart from a broad diffuse scattering bump at small angles. To investigate this further, an additional, third compression to 28 GPa was performed on UiO-66 (Figure S11). This was carried out on the same sample that was used for the first and second compressions.

**Figure 5:**
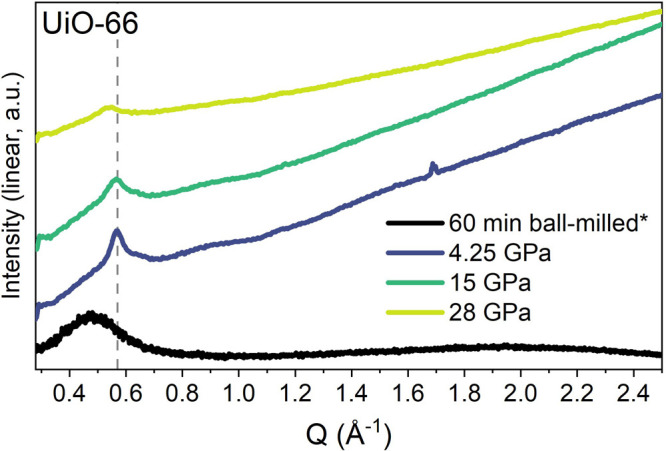
XRD data for UiO-66 after ball-milling for 60 min and at the maximum pressure of each *in situ* hydrostatic compression. Data were taken using either a lab source (asterisk) or a synchrotron. Data were normalized using the most intense Bragg peak (111 reflection) under ambient conditions.

Diffraction patterns from UiO-66 at the maximum pressure of each compression are shown in Figure 5 as a function of *Q*, where *Q* = 4πsinθ/λ. This allows comparison of the synchrotron measurements with a lab-source pattern from a sample fully amorphized by 60 min of ball-milling but measured using a different X-ray wavelength.

At 28 GPa, the XRD data did not contain a feature at the same *Q* position as had been observed at 4.25 and 15 GPa (Figure 5). Instead a broader, lower-intensity feature was observed at a slightly lower *Q* position. While the shape was closer to that of the feature seen in the pattern of the ball-milled amorphous sample, it was still not as broad and the position was dissimilar. Classification of the compressed sample as being truly amorphous as opposed to partially crystalline is difficult, but comparison of the data in Figure 5 implies that UiO-66 was not fully amorphous even after compression to 28 GPa. This comparison was carried out for all MOFs and confirmed the presence of Bragg features in the recovered samples (Figure S12).

The full width at half-maximum (fwhm) of the two most intense Bragg peaks from each MOF was used as an indicative guide to crystallinity (Figure S13). In NU-1000 and MOF-808, the fwhm reaches a clear plateau after an initial increase. Therefore, some crystallinity was lost initially, with the remaining crystallinity surviving even as the pressure exceeded 10 GPa. This is consistent with small-angle Bragg peaks remaining distinct although broad at these pressures. UiO-66 showed a sharper increase in fwhm at low pressures, indicating a larger initial loss of crystallinity.

**Figure 6:**
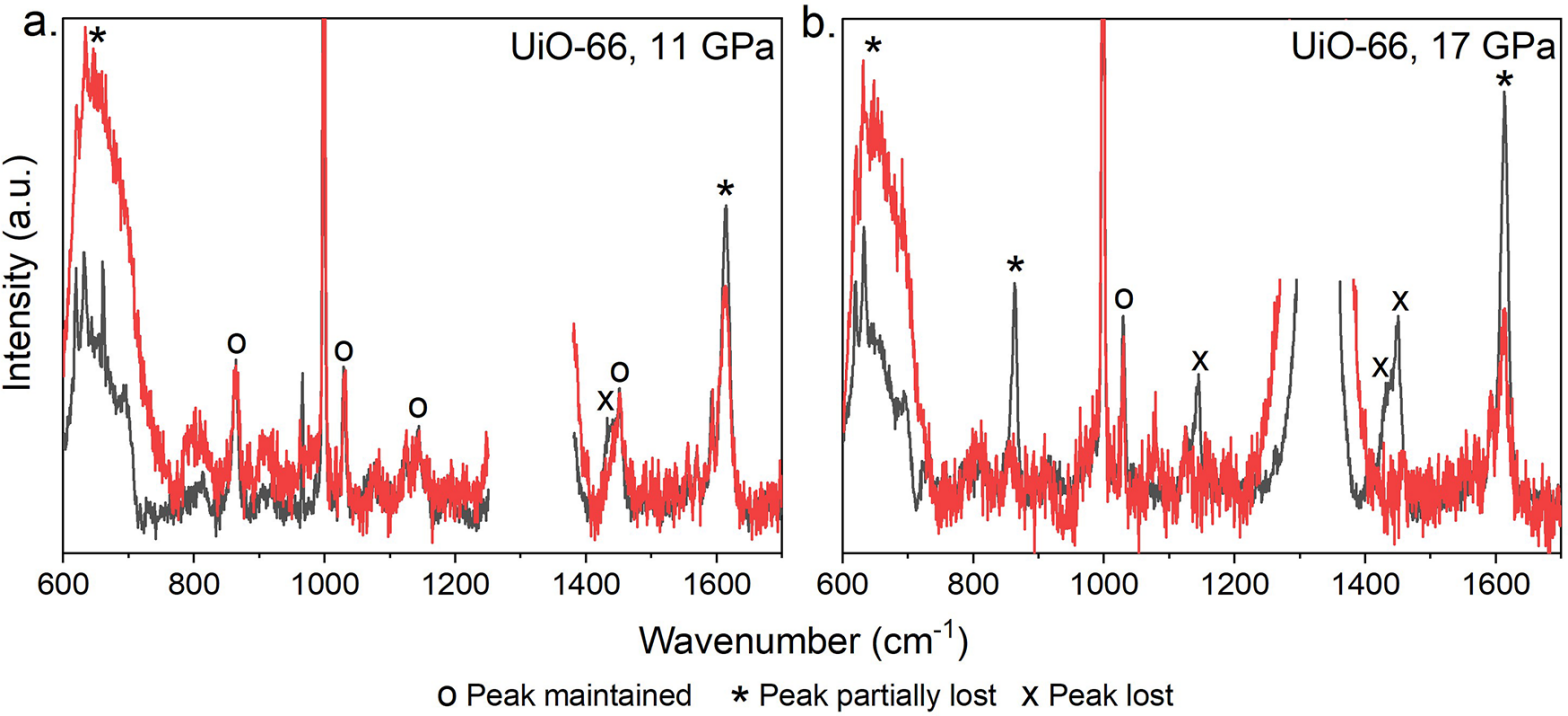
Raman spectroscopy of UiO-66 before compression (black) and after decompression (red) from a maximum pressure of (a) 11 GPa, below the peak-shift threshold, and (b) 17 GPa, above the peak-shift threshold. The data range was selected to avoid the peak at 1300 cm^–1^ corresponding to diamond and prevent saturation of the detector.

To investigate if the onset of the peak shift to smaller angles was related to changes within the local chemical environments of the frameworks, *in situ* Raman spectroscopy was performed and the peaks were assigned (Figures S14–S17 and Table S11). Each MOF was compressed once to a pressure underneath the peak-shift threshold and once to a pressure above it. The data for UiO-66 are shown in Figure 6. After decompression from 11 GPa, only the peak ascribed to the carboxylate OCO symmetric or CC aromatic-to-carboxylate vibration (1430 cm^–1^) disappears, implying some disruption of node–linker bonding.^20^ After 17 GPa, the total loss of the same peak as well as the nearby peak at 1450 cm^–1^ shows eradication of node–linker bonding. Loss of the 863 cm^–1^ peak in the spectra of UiO-66 indicates dehydroxylation of the node.^36^ The other two MOF spectra show no change in node–linker bonding after either compression (Figures S15 and S16).

## Discussion

**Figure 7:**
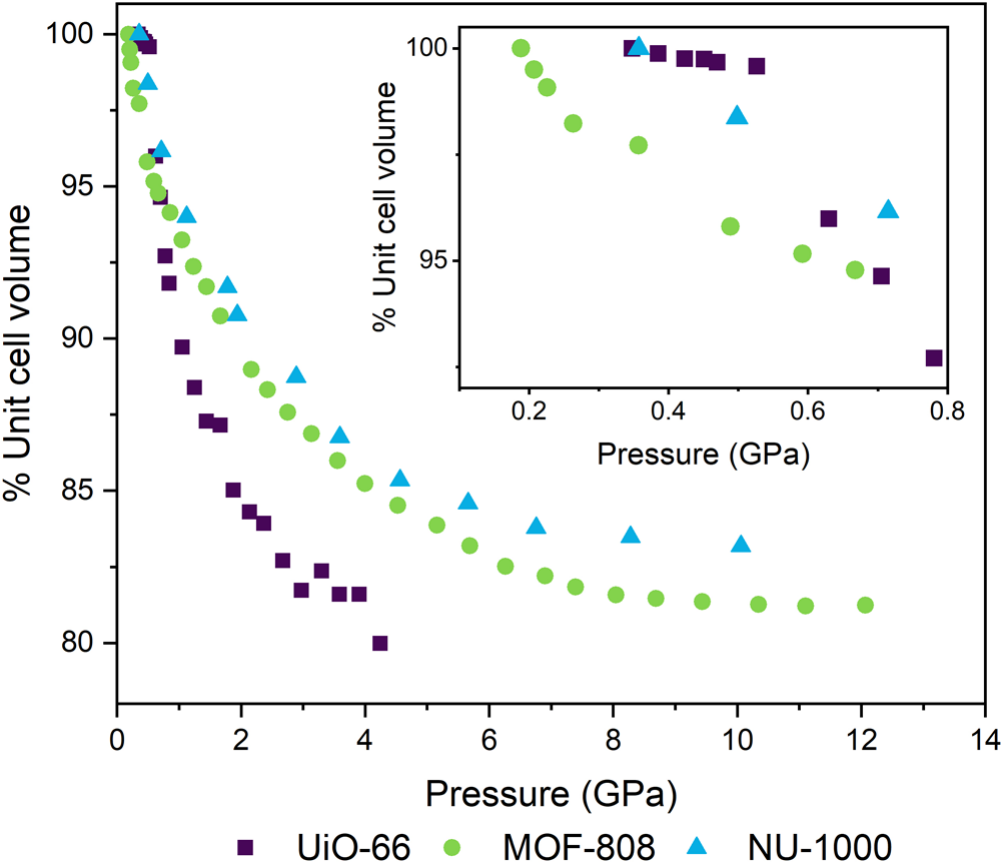
Unit cell volume during quasi-hydrostatic compression of crystalline Zr-MOFs in silicone oil AP100 from Pawley fitting to the initial crystalline phases. The inset shows the low-pressure region of the same data, showing examples of compressibility regime changes in UiO-66 and MOF-808.

To determine whether silicone oil penetrated into the pores of each MOF, their volume change under pressure was considered (Figure 7). Unit cell volumes were derived using Pawley refinement of the known ambient-pressure crystalline phase, noting again that as the Bragg peaks broaden at higher pressures the data cannot be used to tell unambiguously if the structure remains in the same phase. Each MOF displayed several regimes of compressibility that meant the entire data range could not be reliably fit with a single Birch–Murnaghan equation of state (Figures S18–S20). The Ff plots of MOF-808 and NU-1000 show weak correlation in the data, so their initial bulk moduli have been used for comparison of the relative values and not taken to be absolute.

The EOSFit software suite was used to fit the obtained data with Birch–Murnaghan equations of state.^32^ Up to 0.55 GPa, UiO-66 has a bulk modulus of 39.0(4) GPa. This is comparable to the literature value of 41 GPa predicted for an empty framework,^18^ implying no penetration of the silicone oil. The modulus then decreases to 2.7(4) GPa in the 0.55–2.75 GPa region. This decrease shows penetration still has not occurred, as oil being forced into the pores would lower the compressibility and increase the bulk modulus. MOF-808 has an initial bulk modulus of 7.6(3) GPa, increasing to 15(2) GPa for pressures of >0.5 GPa. NU-1000 has an initial bulk modulus of 8.2(2) GPa, whereas the calculated value for a “defect-free” material is 20 GPa.^37^ For pressures above 0.75 GPa, the modulus increased to 16(2) GPa.

UiO-66 would be expected to be the least compressible of the three frameworks because it possesses the highest density and the SBU with the highest connectivity of the three MOFs studied. However, the relative overall volume change (Figure 7) shows the opposite behavior, suggesting that penetration of the PTM likely occurs in both MOF-808 and NU-1000 but not in UiO-66. This is also consistent with the findings of Celeste et al., who demonstrated that silicone oil PDMS (similar to the AP100 used here, but without the phenyl group) was seen to penetrate into MIL-101.^11^ This framework has pore apertures of 12–16 Å, larger than those of UiO-66 but not as large as those of the other two (Table 3).

**Table 3:** Dimensions of the Largest Pore within Each Zr-MOF^a^

MOF	pore aperture diameter (Å)	pore internal diameter (Å)	pore volume (cm^3^/g)	refs
UiO-66 (oct)	6	12–13.8	0.77	(38), (39)
MOF-808 (ad)	14	18.4	0.84	(40), (41)
NU-1000 (hex cyl)	31	31	1.44	(42)

a oct = octahedral, ad = adamantane, and hex cyl = hexagonal cylindrical.

Shifts of peak positions to smaller angles with an increase in pressure after a certain threshold were observed for all three MOFs, with the position of the peak in the XRD pattern of NU-1000 being far more affected than those of the others (Figure 3). The ambient conditions crystalline phase of each MOF could be fit to the respective XRD data measured at every pressure. Assuming that no phase change is taking place, then the refined *a* parameter of UiO-66 and MOF-808 and both *a* and *c* parameters in NU-1000 increase above this pressure threshold (Tables S8–S10). While this phenomenon has been observed in MOFs, it is generally seen at very low pressures when the PTM initially penetrates the crystalline MOF^43,44^ or during partial (zone) collapse where guests are forced from an amorphous region into the crystalline one.^45^ Here, neither rationale seems to be applicable given the already occupied pores of MOF-808 and NU-1000 at the time of the transition. Furthermore, all three MOFs display this apparent ”volume expansion” behavior despite differences in PTM occupation.

However, the assumption that the lattice symmetry does not change must be questioned given the observed discontinuities in the plots of volume versus pressure, as highlighted when refining bulk moduli from the data. At extreme pressures, the values for unit cell volume obtained in this way therefore become unreliable. Instead, the peak shift is likely only due to expansion along one or two axes of the unit cell, and not a true increase in volume. This may be due to nonhydrostaticity within this cell, although a transition purely due to this effect would be expected to take place closer to the PTM’s hydrostatic limit of ∼7 GPa.^27^ It is therefore more likely that this downward peak shift is the result of a structural change effecting the symmetry of any crystalline regions left, for example a change in SBU symmetry similar to that seen at high temperatures,^46^ or breakage of node–linker bonds as seen under nonhydrostatic compression.^47^

The Raman data for MOF-808 and NU-1000 showed no change in the region ascribed to the CO_2_ symmetric stretch (1400–1470 cm^–1^, a Raman-active proxy for the Zr–O node–linker bond^48^) after compression to a pressure at which volume expansion was seen. Therefore, this structural change is not related to bond breakage occurring at these pressures and in these two MOFs may be considered an isostructural phase transition. In UiO-66, however, a decrease in the number of intact node–linker connections was observed with an increase in pressure. Given it was also the only MOF to have no PTM penetration, the bond breakage is therefore ascribed to a lack of reinforcement from pore occupation. Interestingly, UiO-66 still showed the volume expansion behavior.

## Conclusions

In conclusion, a trio of MOFs with the same Zr_6_O_8_ SBU and varying linker identities were investigated under extreme pressure for the first time. Survival of partial crystallinity above 10 GPa was seen in all cases, even in UiO-66 after decompression from 28 GPa. The compressibility of UiO-66 was also seen to be higher than those of MOF-808 and NU-1000. This is attributed to the penetration of the silicone oil PTM into the larger pores of NU-1000 and MOF-808, but not into those of UiO-66.

In each MOF, a clear threshold was seen where the structure underwent a transition from semireversible changes under compression to irreversible changes, which was characterized by an increase in one or more lattice parameters. The structural changes causing this behavior remain unknown but are not accompanied by any changes in bonding. *In situ* X-ray total scattering and spectroscopic measurements are planned to further probe the local order and bonding around the SBU and determine the nature of these changes.

Penetration of the large molecule PTM allowed the survival of partial crystallinity and retention of bonding to high pressures in MOF-808 and NU-1000. Recovery of crystallinity upon decompression was also seen below a certain pressure threshold. This finding provides insight into the pressure response of each MOF. This knowledge is important in applications of these MOFs involving any pressure during processing or use and can be used as a basis for research under conditions more similar to those of a specific utilization.

## Supporting Information Available

Description of synthesis methods, methods and results of additional characterization techniques (X-ray diffraction, gas adsorption, microanalysis, nuclear magnetic resonance, thermogravimetric analysis, and infrared spectroscopy), and high-pressure diffraction and Raman spectroscopy data and their respective analyses (PDF)
