# Supplementary material for: Survival of Zirconium-Based Metal–Organic Framework Crystallinity at Extreme Pressures
Source: Inorg Chem. 2023 Jun 16;62(26):10092–9. doi: 10.1021/acs.inorgchem.2c04428 (PMC10324312; doi:10.1021/acs.inorgchem.2c04428)
Supplement: Supplementary file 1 — ic2c04428_si_001.pdf [file ic2c04428_si_001.pdf]

Survival of Zirconium-based Metal-Organic Framework Crystallinity at Extreme Pressures

Georgina P. Robertson,[†,‡] Sara Mosca,[¶] Celia Castillo-Blas,[†] Florencia Son,[§] Omar K. Farha,[§] David A. Keen,[‖] Simone Anzellini,[‡] and Thomas D. Bennett,[*,†]

† Department of Materials Science and Metallurgy, University of Cambridge, 27 Charles Babbage Road, Cambridge, Cambridgeshire, CB3 0FS, UK

‡ Diamond Light Source Ltd, Diamond House, Harwell Campus, Didcot, Oxfordshire, OX11 0DE, UK

¶ STFC, Rutherford Appleton Laboratory, Harwell Campus, Didcot, Oxfordshire, OX11 0QX, UK

§ Department of Chemistry, Northwestern University, 2145 Sheridan Road, Evanston, IL 60208, USA

‖ ISIS Facility, Rutherford Appleton Laboratory, Harwell Campus, Didcot, Oxfordshire, OX11 0QX, UK

E-mail: tdb35@cam.ac.uk

Table of contents

Section 1: Synthesis **2**

1.1 Materials 2

1.2 Synthesis 2

Section 2: Techniques **2**

Section 3: Characterisation **3**

3.1 Pawley fitting (**Fig. S1, Table S1**) 3

3.2 Nitrogen adsorption analysis (**Table S2, Fig. S2**) 4

3.3 Microanalysis (**Table S3**) 6

3.4 ^{1}H-NMR analysis (**Fig. S3-5, Table S4-6**) 7

3.5 Thermogravimetric Analysis (**Fig. S6, Table S7**) 10

3.6 Fourier Transform Infrared Spectroscopy (**Fig. S7**) 11

Section 4: High pressure measurements **12**

4.1 X-ray diffraction (**Fig. S8-13, Table S8-10**) 12

4.2 Raman Spectroscopy (**Fig. S14-17, Table S11**) 18

4.3 Equation of state fitting (**Fig. S18-20**) 20

References **22**

Section 1: Synthesis

1.1 Materials

$ZrCl_4$ (99.5%), terephthalic acid (98%), $ZrOCl_2 \cdot 8H_2O$ (98%), benzoic acid (99%) and trifluoroacetic acid were purchased from Sigma Aldrich. Hydrochloric acid, N, N-dimethylformamide (DMF, high performance liquid chromatography grade), and acetone (American chemical society grade), were purchased from Fisher Chemical. Methanol (98%) was purchased from Alfa Aesar. $ZrOCl_2 \cdot 8H_2O$ (98%), trimesic acid (98%), and formic acid (98%) were purchased from Acros Organics. 1,3,6,8-tetrakis(p-benzoic acid)pyrene was synthesized based on a published procedure[1] with minor modifications.[2]

1.2 Synthesis

UiO-66 was synthesised using a combination of methods from literature[3,4]: $ZrCl_4$ (0.945 g, 4.06 mmol), terephthalic acid (1.34 g, 8.07 mmol) and hydrochloric acid (0.715 mL) in 24.35 mL of DMF was placed into 50 mL Teflon autoclaves. The solutions were heated to 180°C for 24 hours in an oven. The crystals were then collected *via* centrifugation, washed 2 times with DMF (2 x 20 ml) and solvent exchanged with methanol overnight. They were then desolvated under vacuum at 250°C overnight, then at 300°C for 3 hours. The powder was stored in a desiccator under ambient conditions and evacuated again at the same temperature before compression testing.

MOF-808 was synthesised using a method from literature[5]: Trimesic acid (262 mg 0.480 mmol) and $ZrOCl_2 \cdot 8H_2O$ (407 mg, 1.26 mmol) were mixed in DMF (50 mL) and formic acid (50 mL). The mixture was placed in a 100 mL solvothermal jar, which was heated at 130 °C for 48 hours. The crystals were soaked overnight, washed in DMF, and collected by centrifugation. This was then repeated with methanol. The MOF-808 was activated at 150 °C overnight. The powder was stored in a desiccator under ambient conditions and evacuated again at the same temperature before compression testing.

NU-1000 was synthesised using a method from literature[1]: $ZrOCl_2 \cdot 8H_2O$ (0.776 g, 2.41 mmol) and benzoic acid (16 g, 0.128 mol) in 48 mL of DMF was placed into 250 mL glass bottles. In an 8-dram vial, 1,3,6,8-tetrakis(p-benzoic acid)pyrene (0.320 g, 0.48 mmol) was added to 16 mL of DMF. Both solutions were sonicated for 5 minutes, then placed in a 100 °C oven for 1 hour. The linker solution and trifluoroacetic acid (320 µL, 4.2 mmol) were then added to the bottles, and the mixture sonicated for 5 minutes before placing in a 120 °C oven for 18 hours. The product was isolated by centrifugation and washed three times with DMF, soaking the material for approximately 1 hour between each wash. The powder was suspended in a 100 mL glass bottle with 52 mL DMF and aqueous hydrochloric acid (2 mL, 8 M) and placed in a 100 °C oven overnight. This acid wash was repeated twice. The final product was collected by centrifugation, washed three times with DMF (1 hour soak in between), three times with acetone (1 hour soak in between), and finally soaked in acetone overnight. NU-1000 was collected by centrifugation, dried in an 80 °C vacuum oven for an hour, then thermally activated under dynamic vacuum at 120 °C until an outgas rate of 0.02 mmHg s^{-1} was achieved. The powder was stored in a desiccator under ambient conditions and evacuated again at the same temperature before compression testing.

Section 2: Techniques

Gas Adsorption Measurement: Nitrogen adsorption isotherms were performed on a Micromeritics ASAP 2020 unit. Samples of ~80 mg were degassed under vacuum for 12 hours at 150 °C. Nitrogen isotherms were performed at 77 K. Brunauer-Emmett-Teller (BET) surface area and pore volume distribution were calculated with Micromeritics MicroActive software.

Microanalysis: CHN and Cl combustion analysis was carried out at the Yusuf Hamied Department of Chemistry in Cambridge using a CE440 Elemental Analyser, EAI Exeter Analytical Inc. For each sample, three measurements were taken and averaged.

Nuclear Magnetic Resonance (NMR) Spectroscopy: ~1.5 mg of the MOF was combined with 100-200 µl of D_2SO_4 and then sonicated in DMSO-d_6 (dimethyl sulfoxide). NU-1000 was also heated to ~80 °C to facilitate dissolution. UiO-66 data were acquired on a 400 MHz Bruker Avance III spectrometer, using a QNP probe at 25 °C. MOF-808 and NU-1000 data were acquired on a 500 MHz Bruker Avance III HD spectrometer, using a DCH cryogenically cooled probe with a sample temperature of 25 °C.

Thermogravimetric analysis (TGA): TGA was performed with a simultaneous differential scanning calorimetry (DSC)/TGA thermal analysis (TA) instrument Q600 under an argon flow of 20 mL min^{-1}, with a heating rate of 20 K min^{-1} in an alumina pan. TGA under air was performed with a TA Instruments SDT650, with gas flow 50mL min^{-1}, and a heating rate of 10 K min^{-1}.

Fourier-Transform Infrared (FTIR) Spectroscopy: FTIR spectra were collected from KBr pellets using a Bruker Tensor 27 FTIR spectrometer in transmission mode between 550 and 4000 cm^{-1}. Pellets were prepared by dispersing a small amount of powdered sample in KBr and compressing in a 13 mm diameter pellet die for 10 minutes at 10 tons using a pellet press. A background from a pristine KBr pellet was then subtracted from all spectra prior to analysis. Samples were pelletised at 740 MPa for 10 minutes, and IR spectra were measured with a Bruker Tensor 27 FTIR spectrometer.

Section 3: Characterisation

3.1 Pawley fitting

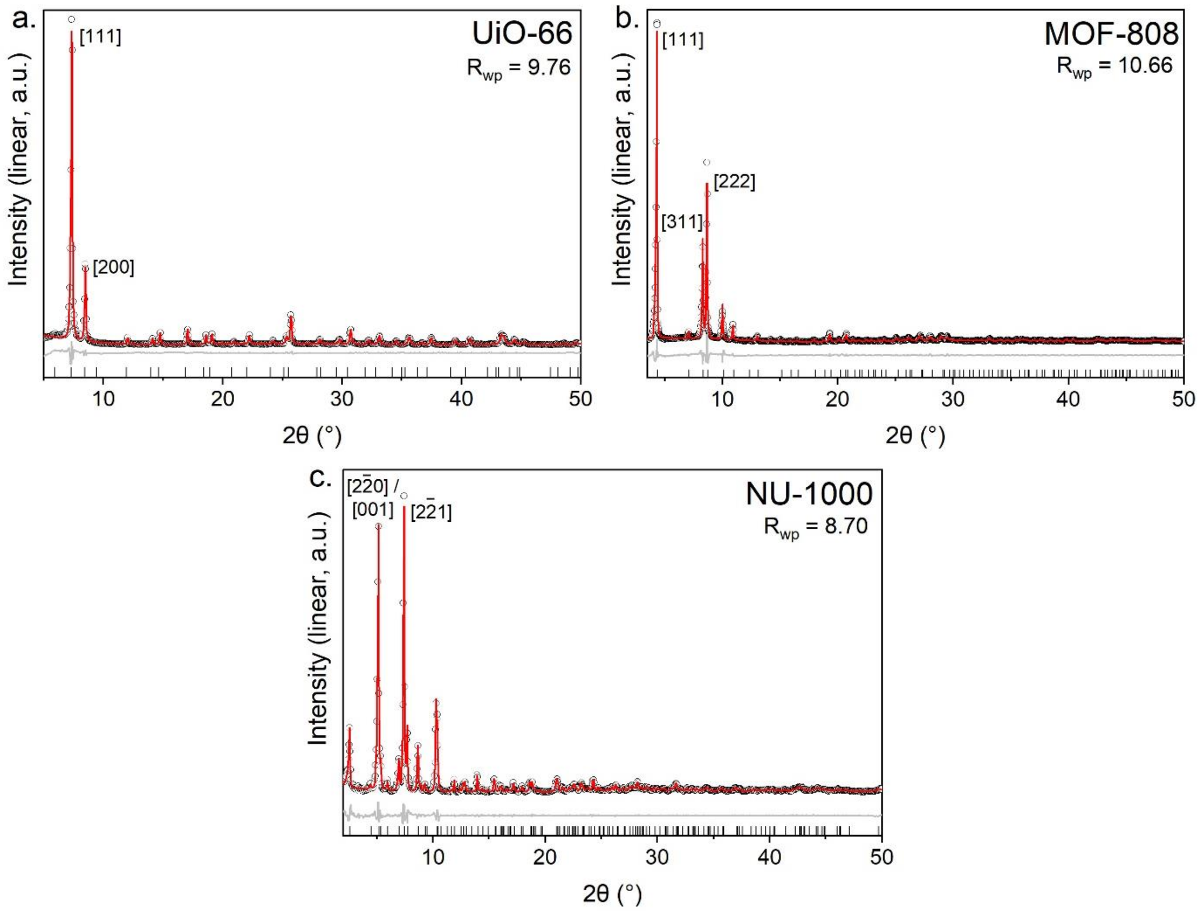

***Figure S1**. Pawley fits of X-ray data for (a) UiO-66, (b) MOF-808 and (c) NU-1000 against a literature CIF using space groups Fm-3m, Fd-3m and P6/mmm respectively (λ = 1.5406 Å).*[6,7] *MOF-808 CIF was calculated by C.C.-B. Initial values of cell parameters were 20.766 Å for UiO-66, 35.308 Å for MOF-808, and a = 39.734 Å, c = 16.506 Å for NU-1000.*

MOF	Lattice parameters from CIF	Calculated lattice parameters
**UiO-66**	a/b/c = 20.7004(2) Å	a/b/c = 20.766(1) Å
	α/β/γ = 90 °	α/β/γ = 90 °
	V = 8870.3(2) $Å^3$	V = 8955(1) $Å^3$
**MOF-808**	a/b/c = 35.0764(10) Å	a/b/c = 35.308(3) Å
	α/β/γ = 90 °	α/β/γ = 90 °
	V = 43156.4(4) $Å^3$	V = 44017(4) $Å^3$
**NU-1000**	a/b = 39.3875(7) Å	a/b = 39.73(2) Å
	c = 16.4829(3) Å	c = 16.506(9) Å
	α/β = 90 °	α/β = 90 °
	γ = 120 °	γ = 120 °
	V = 22145.3(9) $Å^3$	V = 22560(10) $Å^3$

Table S1. *Lattice parameters from Pawley refinement of MOFs compared to literature*

3.2 Nitrogen adsorption analysis

MOF	BET surface area (m^2 g^{-1})	Literature value (m^2 g^{-1}) [2,3,8]
**UiO-66**	931 ± 4	1105-1455
**MOF-808**	1710 ± 90	1140-1390
**NU-1000**	2230 ± 30	2220

Table S2. *BET surface area values compared to those found in literature. Variation is likely due to presence of both defects and remnant excess linker molecules from synthesis. In particular, UiO-66 was determined during elemental analysis to have adsorption of DMF within the pores.*

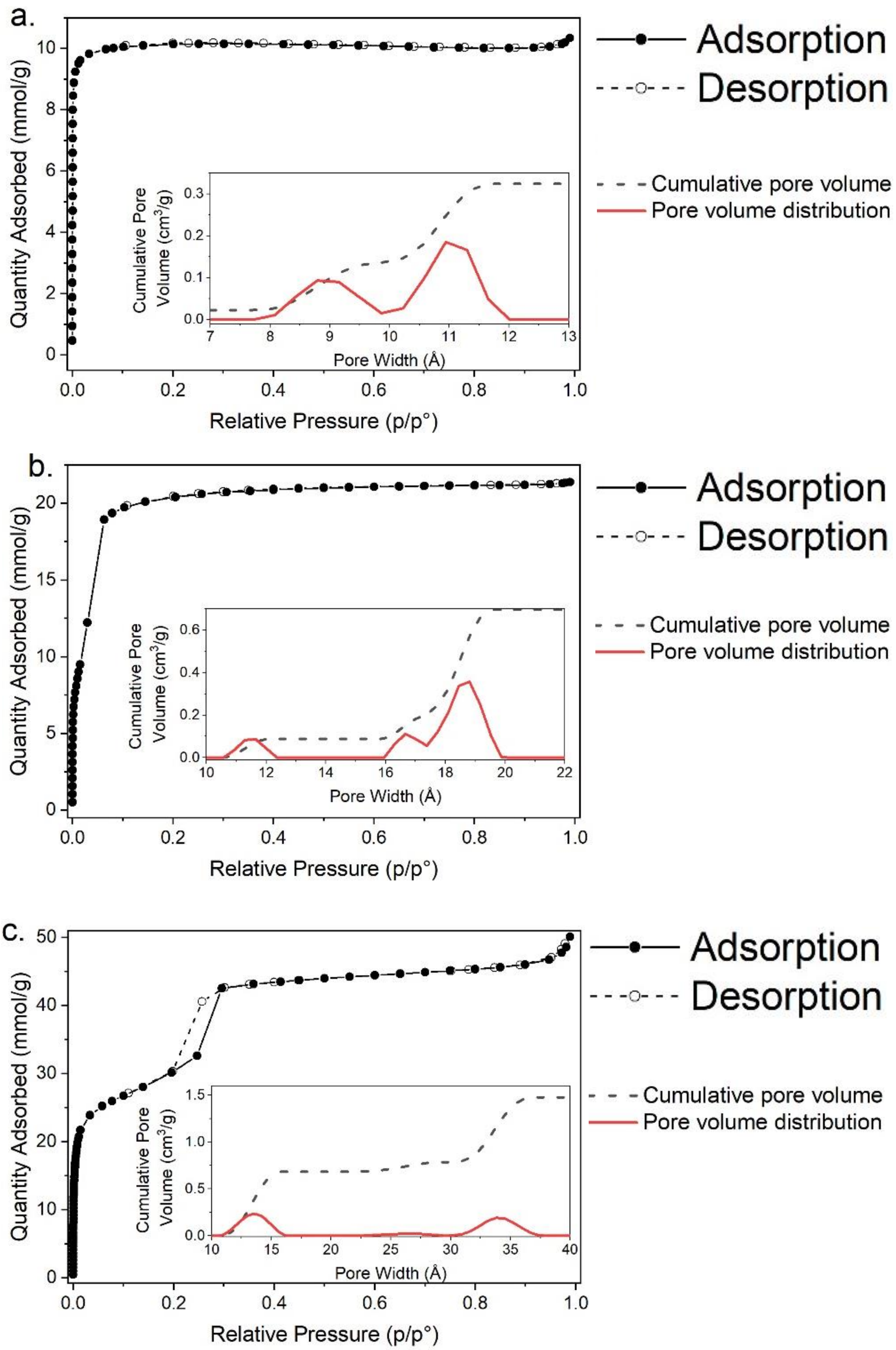

Figure S2. *Nitrogen adsorption isotherms at 77 K and (inset) pore volume distributions of (a) UiO-66, (b) MOF-808 and (c) NU-1000.*

3.3 Microanalysis

UiO-66 - Through ^{1}H-NMR spectroscopy 1,4-BDC was observed in UiO-66 along with trace amounts of DMF (**Fig. S3**). Elemental analysis confirmed the presence of DMF and also showed the presence of Cl^- ions and excess water. TGA showed an average of 5.6 linkers per SBU, leaving 0.8 vacant coordination sites per SBU for potential coordination of the three other species (**Fig. S6, Table S7**). Capping mechanisms have been shown to involve the addition of one -1 ion and one neutral species for every missing linker, with Cl^-/H_2O lowering the free energy more than Cl^-/DMF.[9] Any additional presence of species was compensated for by adding adsorbents. Therefore, the formula most closely fitting the elemental analysis was $Zr_6O_4(OH)_4(OH_2)_{0.8}(C_8H_4O_4)_{5.6}Cl_{0.8}$.DMF, although this underestimates the amount of Cl^- ions. The lower BET surface area value for UiO-66 compared to literature is additional evidence of the adsorption of DMF.

MOF-808 - ^{1}H-NMR spectroscopy and elemental analysis of MOF-808 indicated the presence of Cl^-, formate ions and DMF in addition to the linker, and was used to calculate a formula of $Zr_6O_4(OH)_6(C_9H_3O_6)_2(HCOO)_{1.44}(DMF)_{0.56}Cl_{0.56}$ assuming negligible defects. This agrees with the findings of TGA, which indicates 1.9 linkers per SBU compared to the expected 2 (**Fig. S6, Table S7**). This is within error, and so the assumption of negligible defects is valid.

NU-1000 - ^{1}H-NMR spectroscopy of NU-1000 indicated the presence of 2 DMF molecules for every linker. TGA showed negligible missing linker defects (**Fig. S6, Table S7**), and so these DMF molecules are assumed to be adsorbents. This is confirmed by the low weight percentage of nitrogen found during CHN analysis, as the NU-1000 had been reactivated beforehand, removing the DMF. Elemental analysis also indicates the presence of Cl^- ions from synthesis, and was used to calculate a formula of $Zr_6O_4(OH)_7Cl(OH_2)_4(C_{44}H_{22}O_8)_2$.

	**C**	**H**	**N**	**Cl**
**UiO-66** $Zr_6O_4(OH)_4(H_2O)_{0.8}(C_8H_4O_4)_{5.6}Cl_{0.8}$ .DMF				
**Expected (%)**	33.5	2.06	0.82	1.65
**Actual (%)**	32.6, 26.7	2.01, 2.54	0.68, 1.46	5.85
**MOF-808** $Zr_6O_4(OH)_8(C_9H_3O_6)_2(HCOO)_{1.44}(DMF)_{0.56}$ $Cl_{0.56}$				
**Expected (%)**	19.7	1.52	0.61	1.54
**Actual (%)**	18.47	1.65	0.80	1.85
**NU-1000** $Zr_6O_4(OH)_7Cl(OH_2)_4(C_{44}H_{22}O_8)_2$				
**Expected (%)**	48.15	2.71	0	1.61
**Actual (%)**	47.89	2.36	0.11	1.20

Table S3. *Expected and actual weight percentages of elements within the MOFs. CHN analysis was carried out separately from chlorine. Two values are given where different batches were analysed, to demonstrate variation. Error for this technique is <0.5 wt%.*

3.4 ^{1}H-NMR analysis

UiO-66

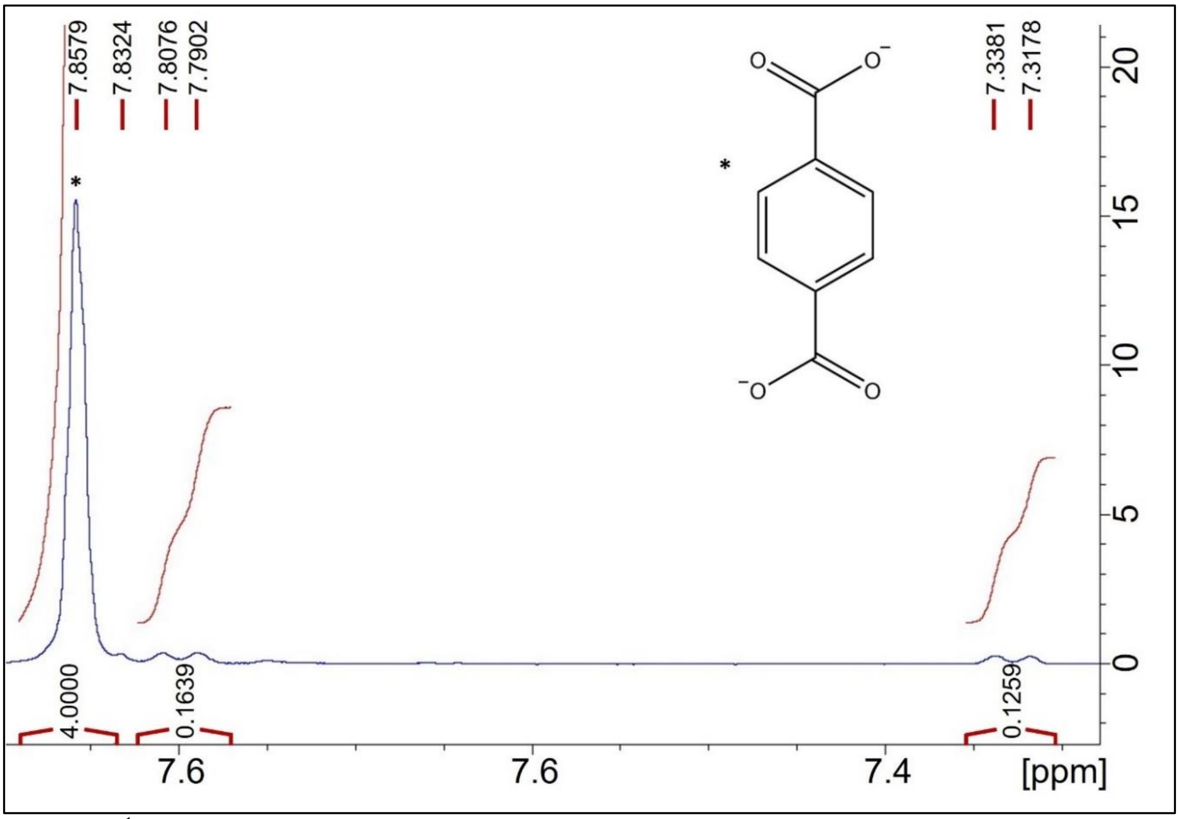

Figure S3. *^{1}H-NMR signal of UiO-66 in DMSO-d$_6$. Spectrum cropped to only show relevant area.*

Assignation	Shift (ppm)	Calculated shift (ppm)	Shape
1,4-benzenedicarboxylate Ar-H	7.8579	8.09	Singlet
DMF H-CO	7.8324	8.02	Singlet
Potential aromatic impurity	7.8076/7.7902	-	Doublet
Potential aromatic impurity	7.3381/7.3178	-	Doublet

Table S4. *NMR peak assignments for UiO-66 using calculated value from Chemdraw Professional.*

MOF-808

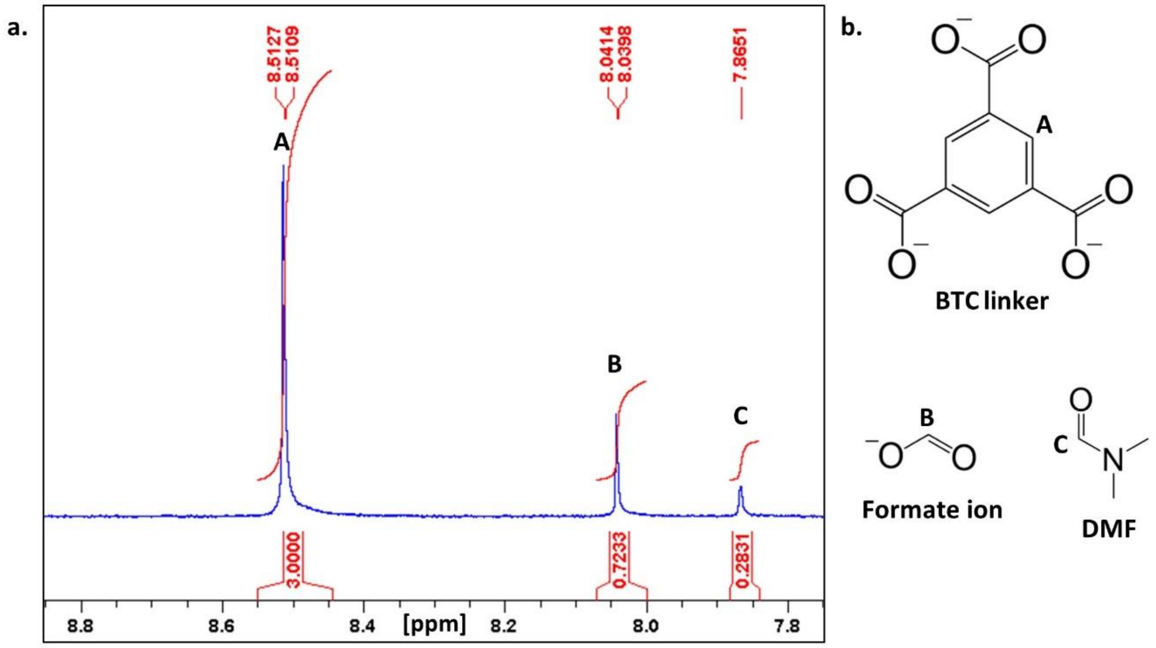

Figure S4. *(a) ^{1}H-NMR signal of MOF-808 in DMSO-d*$_6$*, with the spectrum cropped to only show relevant area (b) Organic molecules present, with their proton environments.*

Assignation	Shift (ppm)	Calculated shift (ppm)	Shape
DMF H-CO	7.8651	8.02	Singlet
Formate ion H-CO	8.0414/8.0398	8.05	Singlet
1,3,5-benzenetricarboxylate Ar-H	8.5127/8.5109	8.89	Singlet

Table S5. *NMR peak assignments for MOF-808 using calculated value from Chemdraw Professional.*

NU-1000

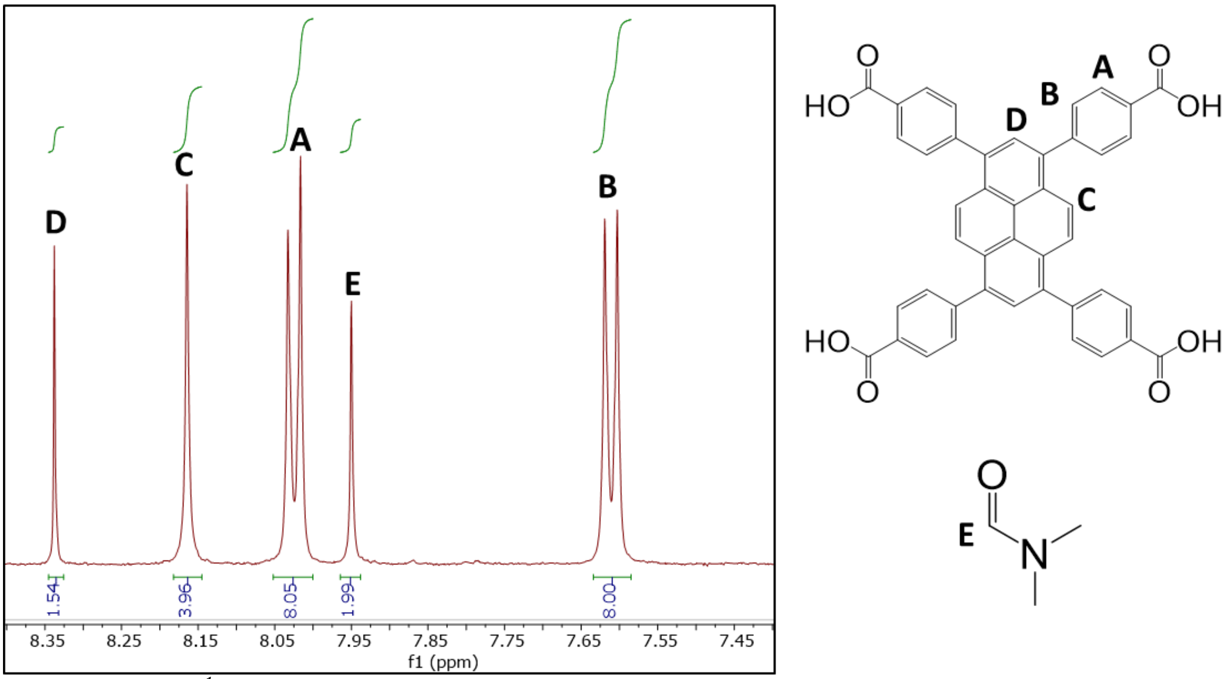

Figure S5. *(a) ^{1}H-NMR signal of NU-1000 in DMSO-d_6 after a single activation and base digestion, with the spectrum cropped to only show relevant area (b) Organic molecules present, with their proton environments.*

Assignation	Shift (ppm)	Calculated shift (ppm)	Shape
TBAPy Ar-H (B)	7.620/7.602	7.85	Doublet
DMF H-CO (E)	7.950	8.02	Singlet
TBAPy Ar-H (A)	8.032/8.015	8.01	Doublet
TBAPy Ar-H (C)	8.163	7.70	Singlet
TBAPy Ar-H (D)	8.363	8.50	Singlet

Table S6. *NMR peak assignments for initial NU-1000 measurement using calculated value from Chemdraw Professional.*

3.5 Thermogravimetric Analysis

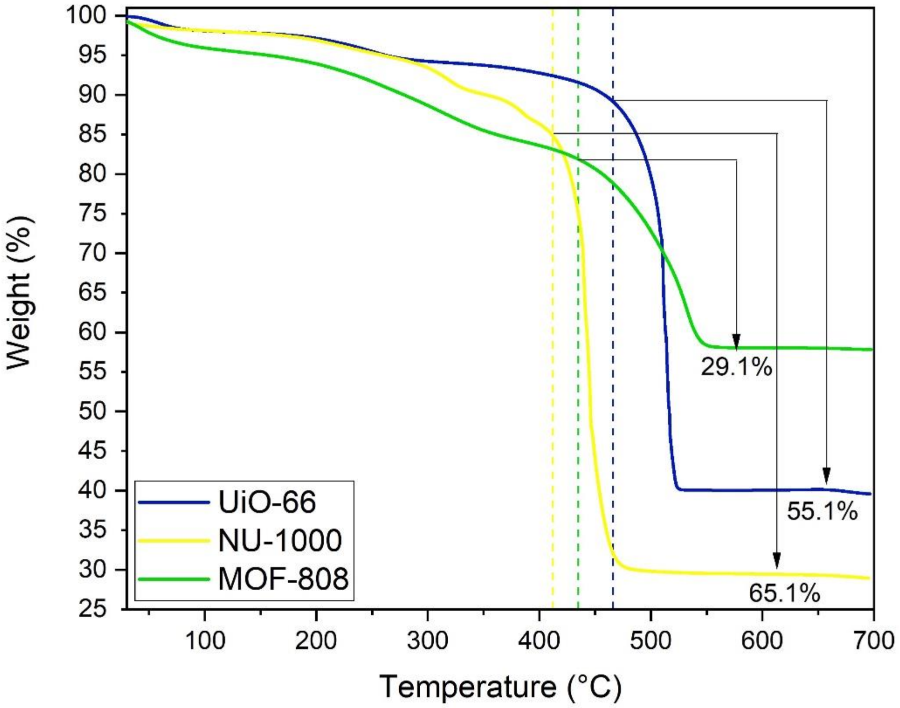

Figure S6. *Weight change under heating in air. Decomposition temperatures shown by dotted lines were calculated by onset of peak in differential. Mass loss shown with reference to initial structure.*

MOF	Hydrated Formula	Dehydrated Formula	Mass loss (theo.)	Mass loss (exp.)	Linkers per node
UiO-66	$Zr_6O_4(OH)_4(C_8H_4O_4)_6$	$Zr_6O_6(C_8H_4O_4)_6$	59.2 %	55.1 %	5.6 (6)
MOF-808	$Zr_6O_4(OH)_4(C_9H_3O_6)_2(HCOO)_6$	$Zr_6O_9(C_9H_3O_6)_2$	30.4 %	29.1 %	1.9 (2)
NU-1000	$Zr_6O_4(OH)_5(OH_2)(C_{44}H_{22}O_8)_2(HCOO)_3$	$Zr_6O_8(C_{44}H_{22}O_8)_2$	61.5 %	65.1 %	2.1 (2)

Table S7. *Calculated linker ratio from mass loss during heating under air, based on the method used by Shearer et al.*[10] *The initial MOF is assumed to be entirely hydrated apart from the formates ions indicated by NMR, the 400 °C phase is dehydrated and has lost formates,*[11] *and a decomposition product of* ZrO_2*. Oxygen compensation was used to ensure charge neutrality for dehydrated MOF-808 and NU-1000, as this reduces error in the method.*[12] *Theoretical mass loss for decomposition of linkers in perfect pristine crystal given.*

Example calculation: UiO-66

$M_w[Zr_6O_4(OH)_4(C_8H_4O_4)_6]$ = 1664.06 amu, $M_w[bdc^{2-}]$ = 164.11

Mass % per linker = $\frac{164.11}{1664.06}$ = 9.86 %

Linkers per node = $\frac{55.1}{9.86}$ = 5.587 = 5.6 linkers

3.6 Fourier Transform Infrared Spectroscopy

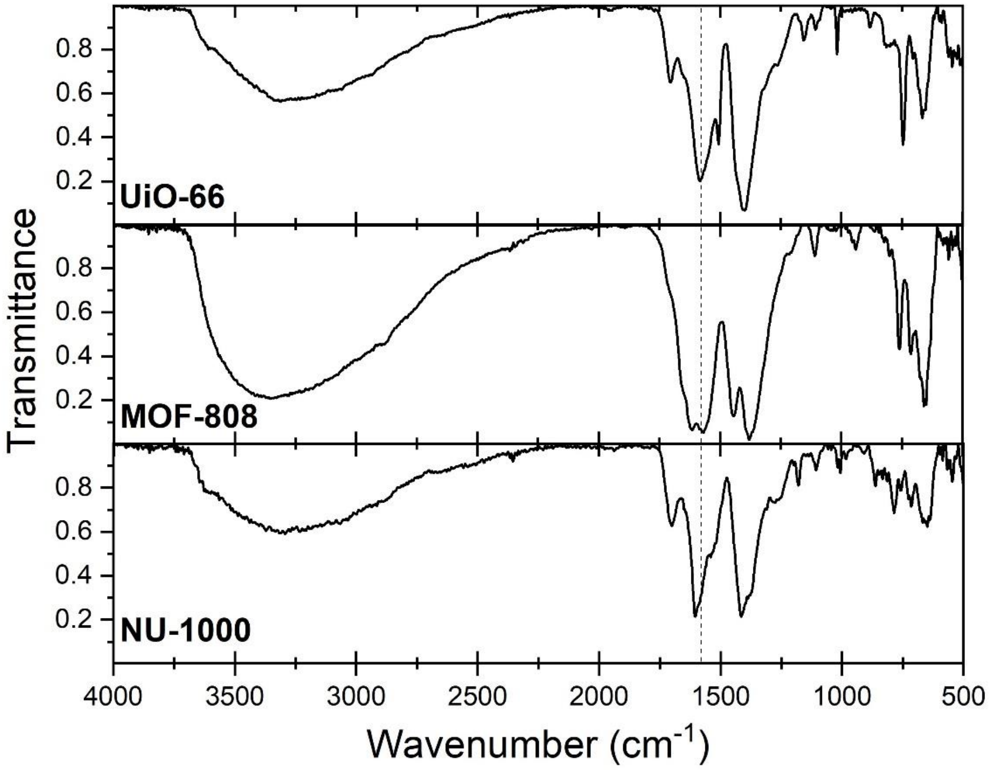

Figure S7. *FTIR spectroscopy data on all three crystalline MOFs. Dashed line indicates 1578 cm^{-1}.*

Each spectrum shows a peak at 1390 cm^{-1} is from the symmetric carboxylate stretch, and at 1578 cm^{-1} from the asymmetric. The shoulder at 1550 cm^{-1} on the latter peak in all MOFs indicates a monodentate carboxylate, which is likely from the pelletisation procedure beforehand.[13,14]

Section 4: High pressure measurements

4.1 X-ray diffraction

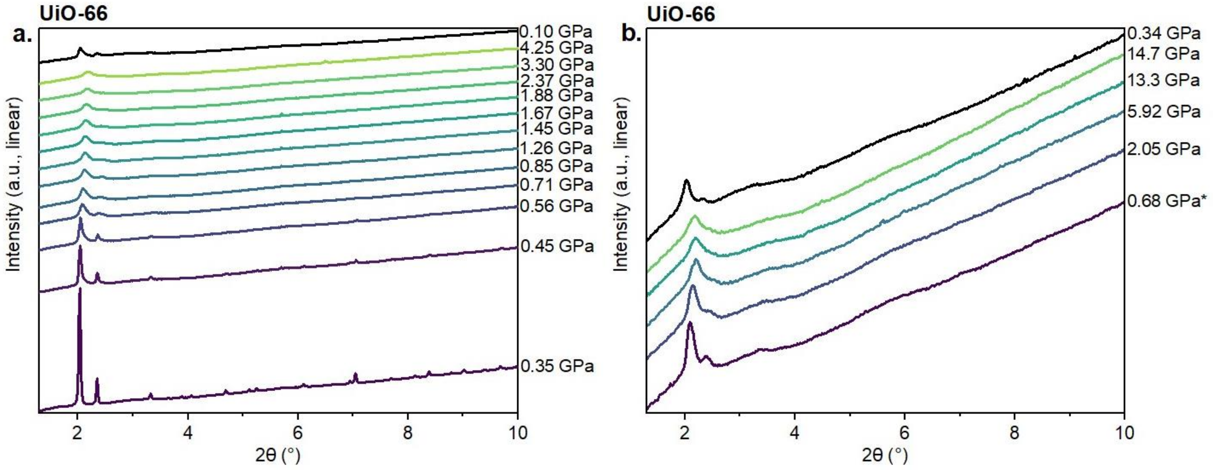

Figure S8. *Synchrotron XRD data under compression for (a) UiO-66 (b) UiO-66 after a previous compression to 4.25 GPa. Post-decompression data after each compression are shown in black at the top. * indicates that while the sample has undergone a previous compression.*

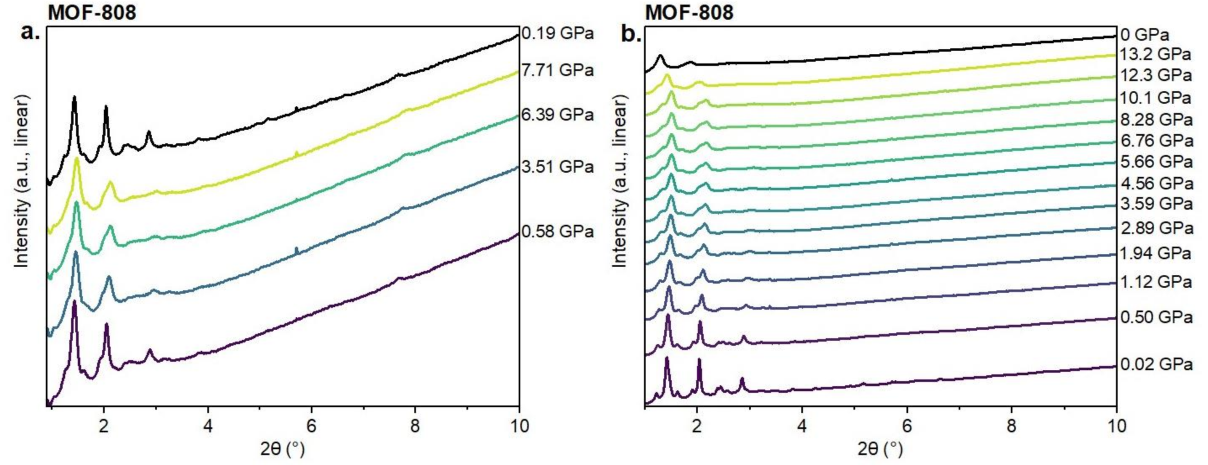

Figure S9. *Synchrotron XRD data under compression for (a) MOF-808 (b) MOF-808 after a previous compression to 12.1 GPa. Post-decompression data after each compression are shown in black at the top. * indicates that while the sample has undergone a previous compression.*

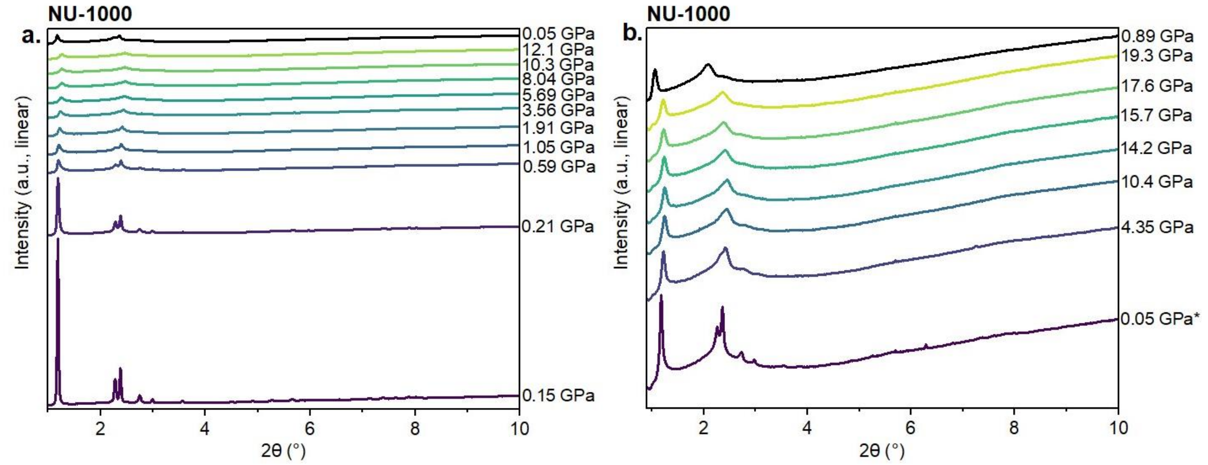

Figure S10. *Synchrotron XRD data under compression for NU-1000 to a maximum pressure of (a) 7.71 GPa and (b) 13.2 GPa. Post-decompression data after each compression are shown in black at the top.*

UiO-66

Filename	R_{wp}	Pressure (GPa)	a (Å)	Error in a (Å)	V (Å^3)	Error in V (Å^3)	ε (left, x10^{-3})	ε error (left, x10^{-3})	ε (right, x10^{-3})	ε error (right, x10^{-3})
339066	0.698	0.35	20.685	0.0008	8850.9	1.1	7.58	0.08	7.46	0.06
339067	0.606	0.39	20.677	0.0009	8839.7	1.1	7.43	0.08	8.15	0.06
339068	0.511	0.42	20.669	0.0010	8829.6	1.3	7.27	0.08	10.0	0.08
339069	0.421	0.45	20.668	0.0014	8828.7	1.8	7.45	0.12	15.3	0.14
339070	0.408	0.47	20.663	0.0019	8822.2	2.4	7.71	0.14	18.2	0.17
339071	0.426	0.53	20.656	0.0024	8813.4	3.1	7.94	0.17	21.9	0.23
339084	0.519	0.63	20.405	0.0074	8495.9	9.2	19.5	0.58	45.1	0.74
339085	0.485	0.71	20.309	0.0075	8376.0	9.3	22.7	0.63	46.0	0.77
339086	0.516	0.78	20.170	0.0053	8205.2	6.5	27.1	0.69	44.8	0.82
339087	0.372	0.85	20.104	0.0035	8125.7	4.2	29.8	0.52	45.8	0.62
339088	0.365	1.06	19.950	0.0050	7939.9	5.9	35.3	0.67	46.3	0.68
339089	0.411	1.26	19.851	0.0076	7822.7	9.0	35.7	0.87	46.2	0.84
339090	0.359	1.45	19.768	0.0052	7725.2	6.1	39.5	0.81	49.1	0.80
339091	0.412	1.67	19.758	0.0069	7713.4	8.0	37.3	0.95	52.6	1.01
339092	0.418	1.88	19.595	0.0102	7524.2	11.7	45.7	1.28	49.0	1.10
339093	0.487	2.14	19.540	0.0121	7460.6	13.9	47.2	1.55	50.7	1.34
339094	0.355	2.37	19.511	0.0090	7427.7	10.3	50.7	1.25	54.9	1.10
339095	0.314	2.67	19.416	0.0069	7319.7	7.9	52.9	1.12	53.1	0.95
339096	0.466	2.98	19.339	0.0110	7232.6	12.4	54.0	1.77	50.9	1.41
339097	0.359	3.30	19.390	0.0077	7289.6	8.7	53.3	1.36	58.1	1.26
339098	0.354	3.59	19.329	0.0097	7221.4	10.8	57.5	1.53	58.7	1.31
339099	0.343	3.91	19.329	0.0085	7221.6	9.6	55.3	1.46	63.4	1.40
339100	0.345	4.25	19.200	0.0070	7078.2	7.8	62.0	1.61	59.7	1.37
339101	0.415	4.18	19.192	0.0123	7068.9	13.6	59.0	1.97	56.6	1.62
339102	0.530	4.00	19.208	0.0168	7086.4	18.6	55.7	2.42	56.2	2.06
339103	0.374	3.65	19.263	0.0023	7148.2	2.5	53.1	1.40	59.1	1.35
339104	0.338	2.46	19.255	0.0041	7138.5	4.5	43.9	1.02	55.1	1.09
339105	0.482	0.06	20.467	0.0050	8573.8	6.2	18.1	0.64	35.0	0.85
339106	0.503	0.10	20.676	0.0033	8839.1	4.3	13.0	0.45	36.0	0.77
339115	0.541	0.68	20.100	0.0053	8120.6	6.4	36.2	1.01	45.0	1.02
339116	0.460	2.05	19.708	0.0081	7654.8	9.4	51.8	1.63	62.7	1.64
339117	0.537	5.92	19.155	0.0127	7027.7	14.0	62.8	2.83	61.8	2.47
339118	0.612	13.27	19.342	0.0182	7235.6	20.4	75.0	4.85	86.8	5.24
339119	0.787	14.69	19.339	0.0097	7232.3	10.9	75.0	5.87	75.0	5.44
339120	0.486	0.34	20.695	0.0118	8863.4	15.1	60.5	2.57	47.4	1.80

Table S8. *Unit cell parameters of UiO-66 derived from Pawley analysis, using LaB_6 standard for peak shape determination, and Lorentzian fitting of each peak's side to determine right-hand and left-hand components of microstrain. Pressure was determined from Pawley fitting of a tungsten calibrant to retrieve lattice parameters, and then equation of state fitting.[15] UiO-66 forms a cubic unit cell in the space group Fm-3m.*

MOF-808

File-name	R_{wp}	Pressure (GPa)	a (Å)	V (Å^3)
339207	2.032	0.19	35.283	43924.40
339209	1.143	0.21	35.224	43704.22
339210	0.966	0.23	35.174	43518.20
339211	0.889	0.26	35.074	43145.70
339212	0.866	0.36	35.013	42923.70
339213	0.852	0.49	34.783	42082.90
339214	0.839	0.59	34.705	41800.00
339215	0.812	0.67	34.658	41632.00
339216	0.700	0.86	34.579	41347.00
339217	0.671	1.05	34.470	40955.00
339218	0.672	1.24	34.361	40570.80
339219	0.667	1.45	34.280	40281.20
339220	0.659	1.67	34.158	39854.70
339221	0.654	1.91	34.062	39520.30
339222	0.642	2.17	33.937	39087.60
339223	0.635	2.43	33.852	38793.40
339224	0.622	2.75	33.758	38469.40
339225	0.656	3.14	33.666	38157.40
339226	0.646	3.56	33.552	37771.00
339227	0.602	4.00	33.454	37439.80
339228	0.577	4.53	33.359	37121.70
339229	0.560	5.16	33.274	36839.20
339230	0.549	5.69	33.184	36540.60
339231	0.548	6.27	33.094	36243.80
339232	0.531	6.90	33.052	36107.00
339233	0.543	7.40	33.003	35945.50
339234	0.506	8.04	32.969	35834.70
339235	0.504	8.69	32.953	35782.30
339236	0.497	9.43	32.939	35737.40
339237	0.477	10.35	32.926	35695.60
339238	0.458	11.10	32.918	35669.80
339239	0.473	12.07	32.921	35680.90
339240	0.446	9.96	33.110	36297.60
339241	0.584	4.64	33.813	38658.70
339242	0.618	0.05	35.388	44315.10
339248	0.778	4.35	34.258	40206.50
339249	0.570	9.84	33.671	38173.00
339250	0.541	10.40	33.611	37970.70
339251	0.416	11.43	33.559	37794.60
339252	0.503	14.19	33.545	37745.40
339253	0.439	15.43	33.637	38059.30
339256	0.420	16.88	33.995	39286.40
339257	0.487	17.61	34.062	39518.50
339258	0.401	19.28	34.294	40331.50

Table S9. *Unit cell parameters of MOF-808 derived from Pawley analysis, using standard TCZH peak fitting. No Lorentzian microstrain component was used here, as unlike the other samples this was found*

to worsen the fitting. Pressure was determined from Pawley fitting of a tungsten calibrant to retrieve lattice parameters, and then equation of state fitting.[15] *MOF-808 forms a cubic unit cell in the space group Fd-3m. Errors in a were ≤ 0.05 %, errors in V were ≤ 0.1 %.*

NU-1000

File-name	R_{wp}	Pressure (GPa)	a (Å)	Error in a (Å)	c (Å)	Error in c (Å)	V (Å^3)	Error in V (Å^3)	ε (left, x10^{-3})	ε error (left, x10^{-3})	ε (right, x10^{-3})	ε error (right, x10^{-3})
339157	0.435	0.36	39.325	0.012	16.485	0.007	22077	16	17.8	0.3	16.2	0.5
339158	0.413	0.50	39.075	0.013	16.425	0.008	21718	18	21.2	0.4	17.0	0.5
339160	0.378	0.71	38.711	0.017	16.358	0.012	21229	24	25.6	0.5	21.0	0.7
339161	0.380	1.12	38.419	0.022	16.236	0.015	20754	30	29.5	0.7	23.6	0.9
339162	0.365	1.78	38.091	0.025	16.110	0.016	20244	33	33.7	0.8	23.6	1.0
339163	0.258	1.94	37.956	0.019	16.061	0.012	20039	25	36.3	0.6	24.8	0.8
339164	0.252	2.89	37.688	0.019	15.927	0.013	19593	26	38.7	0.7	26.3	0.9
339165	0.225	3.59	37.414	0.018	15.801	0.012	19155	23	42.6	0.8	27.8	0.8
339166	0.214	4.56	37.222	0.018	15.702	0.015	18840	26	46.0	0.9	29.3	1.0
339167	0.220	5.66	37.121	0.024	15.650	0.016	18676	30	48.3	1.2	30.7	1.2
339168	0.208	6.76	37.015	0.023	15.588	0.015	18496	29	50.8	1.2	31.5	1.2
339169	0.207	8.28	36.966	0.022	15.575	0.017	18432	30	52.4	1.3	31.8	1.2
339170	0.185	10.06	36.938	0.021	15.542	0.015	18365	27	53.4	1.2	31.6	1.2
339171	0.221	12.26	37.063	0.024	15.585	0.019	18541	33	51.8	1.4	29.7	1.4
339172	0.298	12.51	38.005	0.046	15.910	0.035	19902	65	56.2	2.7	32.1	2.9
339173	0.165	13.25	39.207	0.026	16.318	0.025	21723	44	57.1	1.9	33.9	2.3
339174	0.210	12.11	39.714	0.037	16.436	0.034	22451	63	53.2	2.4	35.5	3.3
339175	0.387	8.94	40.397	0.064	16.449	0.084	23248	139	51.0	5.2	38.6	4.8
339176	0.235	5.38	41.195	0.045	16.802	0.065	24693	110	49.7	4.3	38.3	3.5
339177	0.298	3.50	42.149	0.061	17.163	0.101	26405	173	49.8	6.4	37.9	4.5
339178	0.302	-0.04	43.272	0.085	17.691	0.151	28687	270	51.4	6.7	40.1	6.3
339190	0.787	0.58	39.350	0.065	16.482	0.051	22103	100	32.2	2.6	22.9	3.9
339191	0.680	3.51	39.315	0.150	16.489	0.087	22073	205	38.7	5.5	31.6	3.7
333192	0.617	6.39	39.163	0.187	16.438	0.127	21835	268	47.3	9.4	32.7	4.5
333193	0.556	7.71	39.096	0.179	16.426	0.121	21744	255	48.5	9.6	35.8	3.8
333194	0.647	0.19	39.537	0.050	16.618	0.032	22496	71	25.8	1.6	21.0	2.1

Table S10. *Unit cell parameters of NU-1000 derived from Pawley analysis, using LaB_6 standard for peak shape determination, and Lorentzian fitting of each peak's side to determine right-hand and left-hand components of microstrain. Pressure was determined from Pawley fitting of a tungsten calibrant to retrieve lattice parameters, and then equation of state fitting.*[15] *NU-1000 forms a hexagonal unit cell in the space group P6/mmm.*

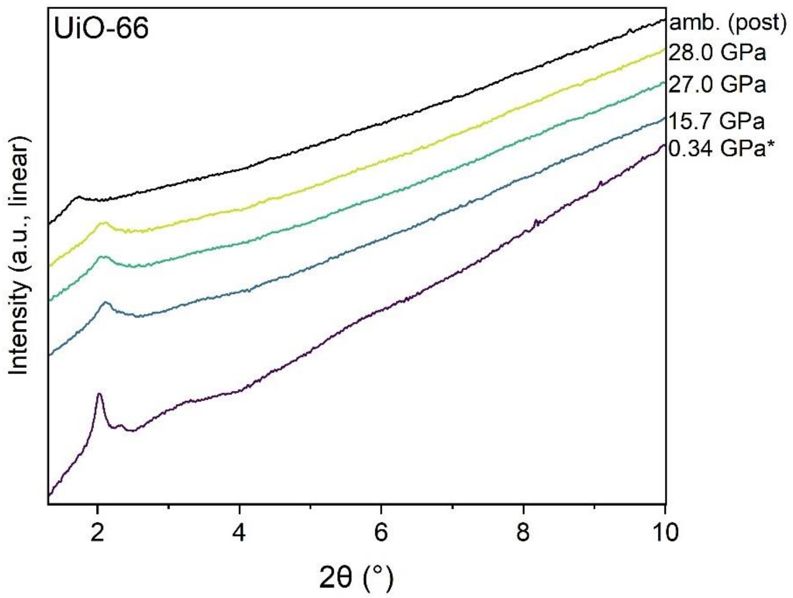

Figure S11. *Synchrotron XRD data under compression for UiO-66 after two previous compressions to 4.25 and then 14.7 GPa. Post-decompression data are shown in black at the top. * indicates that while the sample is at ambient conditions, it has undergone a previous compression.*

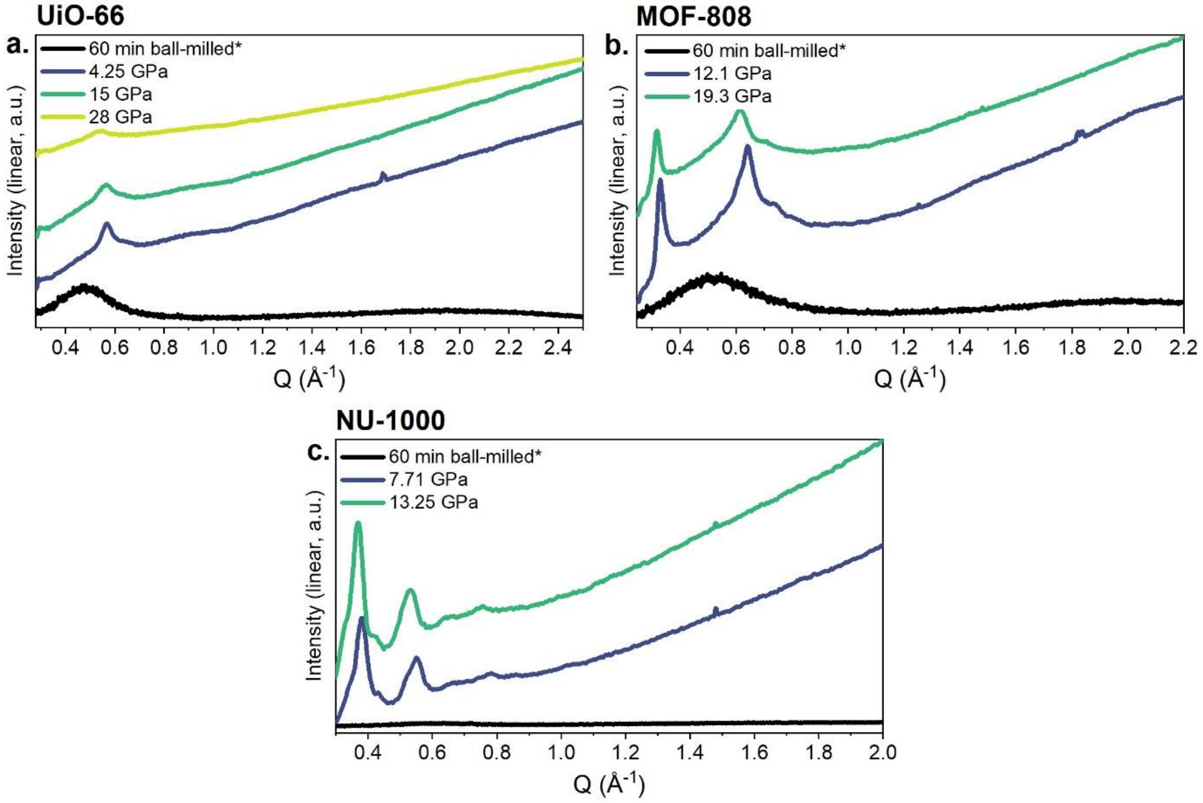

Figure S12. *XRD data for (a) UiO-66, (b) MOF-808, and (c) NU-1000 comparing the MOF after 60 minutes of ball-milling, and MOF at the maximum pressure of each in situ hydrostatic compression. Data were taken using either a lab-source (*) or synchrotron. Synchrotron data were normalised for comparison through multiplication by a factor of 25, 18 and 130 for UiO-66, MOF-808 and NU-1000 respectively. This factor was calculated from equalising the intensities of the most intense Bragg peak in the lab source and synchrotron datasets (the 111 reflection for UiO-66 and MOF-808, and the $2\bar{2}0$ for NU-1000).*

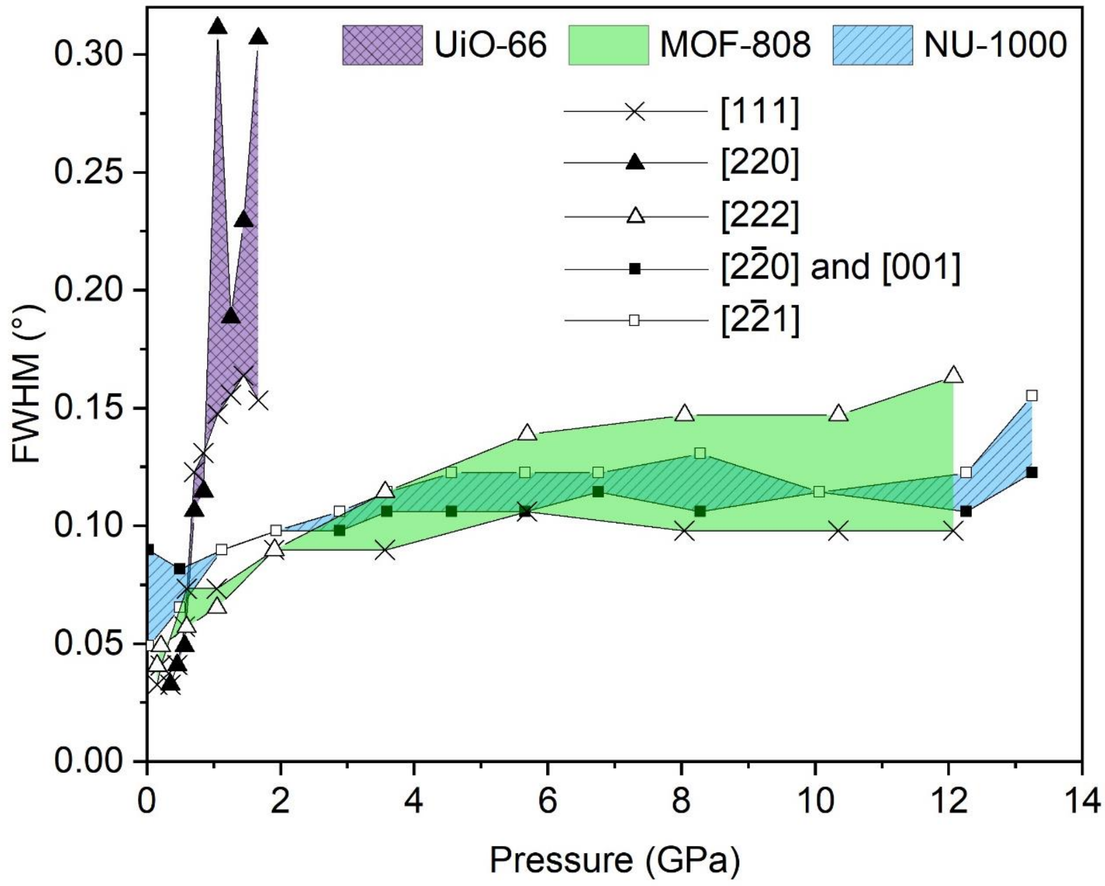

Figure S13. *Variation of the two most intense Bragg peak's FWHMs under pressure for each MOF, with the area between them shaded. All peaks were fitted with a pseudo-Voigt function until a pressure at which the two peaks were no longer distinguishable by the Fityk software used.*

4.2 Raman Spectroscopy

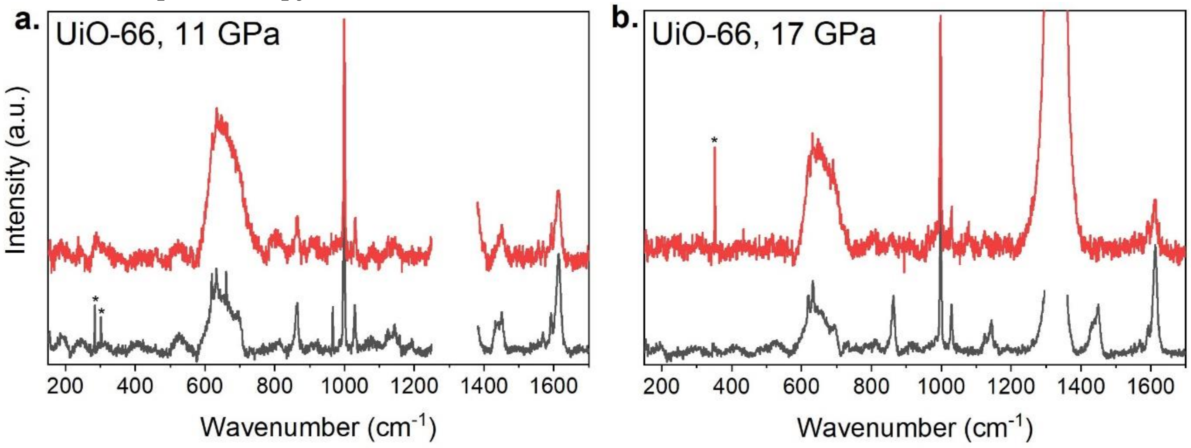

Figure S14. *Raman spectroscopy of UiO-66 before compression (black) and after decompression (red) from a maximum pressure of (a) below the reversibility threshold and (b) above the threshold. Maximum pressure the sample was brought to is shown in the top right. 1300-1350 cm^{-1} range excluded to remove saturating diamond signal.*

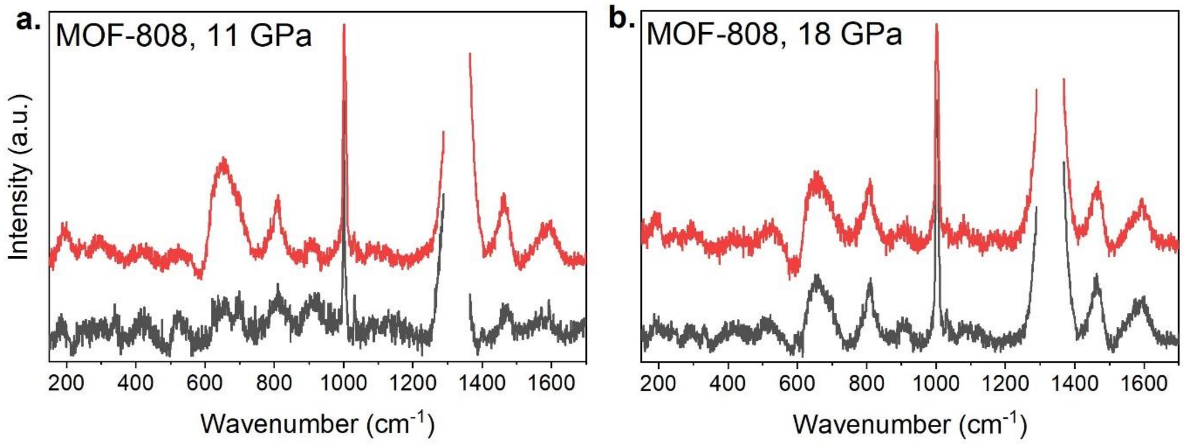

Figure S15. *Raman spectroscopy of MOF-808 before compression (black) and after decompression (red) from a maximum pressure of (a) below the reversibility threshold and (b) above the threshold. Maximum pressure the sample was brought to is shown in the top right. 1300-1350 cm^{-1} range excluded to remove saturating diamond signal.*

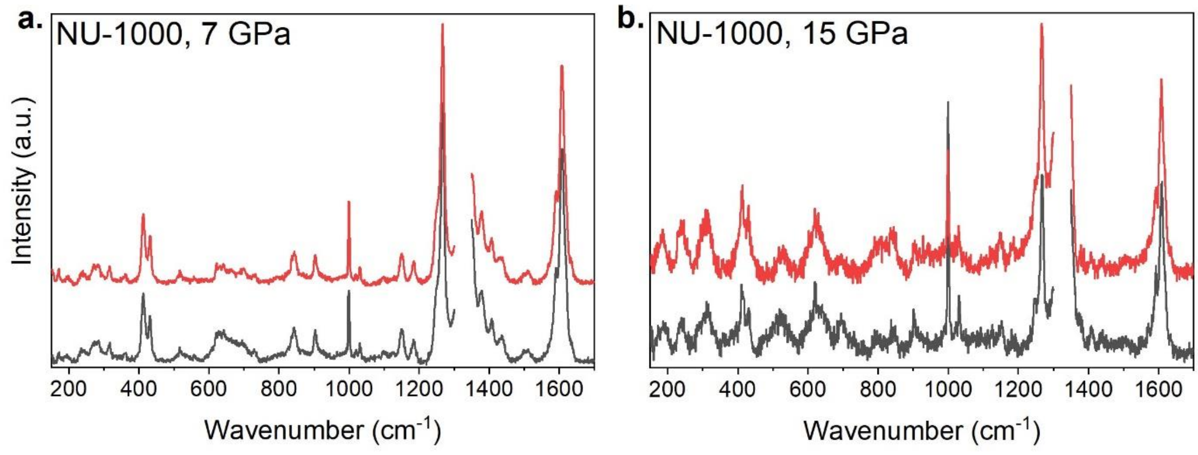

Figure S16. *Raman spectroscopy of NU-1000 before compression (black) and after decompression (red) from a maximum pressure of (a) below the reversibility threshold and (b) above the threshold. Maximum pressure the sample was brought to is shown in the top right. 1300-1350 cm^{-1} range excluded to remove saturating diamond signal.*

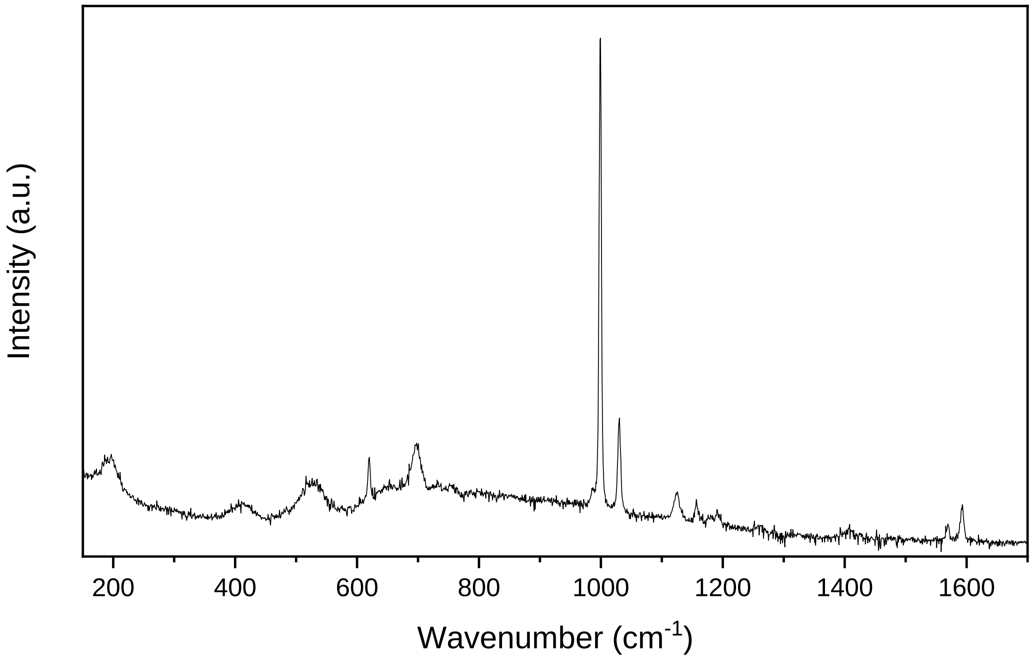

Figure S17. *Raman spectroscopy of a Silicone Oil AP100 droplet on a glass slide under ambient conditions.*

MOF	Vibration frequency (cm^{-1})	Assignment
All	185	Cluster torsion mode with contribution from silicone oil
UiO-66, NU-1000	240	Zr-μ_3O asymmetric stretching?
All	310	Out of plane, out of phase CCC bending
NU-1000	412, 431, 840, 903, 1184	Various TBAPy linker vibrations
All	520	CSi stretch from silicone oil
All	~650 (broad)	CC aromatic ring in place bending + CSi/SiC_2 stretches from silicone oil
All	863	OH bending of MOF's node (+ CC symmetric breathing)
All	1000	Silicone oil aromatic CH out of plane bending
All	1028	Silicone oil aromatic CH out of plane bending
UiO-66, NU-1000	1145	CC symmetric ring breathing
NU-1000	1268	Unknown
All	~1350	Diamond
NU-1000	1378	Unknown

NU-1000	1408	CO_2 symmetric or CC aromatic-to-carboxylate stretch?
UiO-66	1430	CO_2 symmetric or CC aromatic-to-carboxylate stretch
NU-1000	1435	CO_2 symmetric or CC aromatic-to-carboxylate stretch
UiO-66	1450	CO_2 symmetric or CC aromatic-to-carboxylate stretch
MOF-808	1465	CO_2 symmetric and CC aromatic-to-carboxylate stretch
All	1612	CC aromatic in phase stretch with shoulder at 1594 cm^{-1} from silicone oil

Table S11. *List of peak assignments for all Raman spectra, in wavenumber order.*[3,16,17]

4.3 Equation of state fitting

As stated in the main text, fitting of the entire data range was not possible with a single equation of state. Therefore, the first regime was selected to give an initial bulk modulus to compare with the literature and the second to give the relative compressibility for comparison between the MOFs. Regimes were selected through observation of discontinuities in the volumetric compressibility.

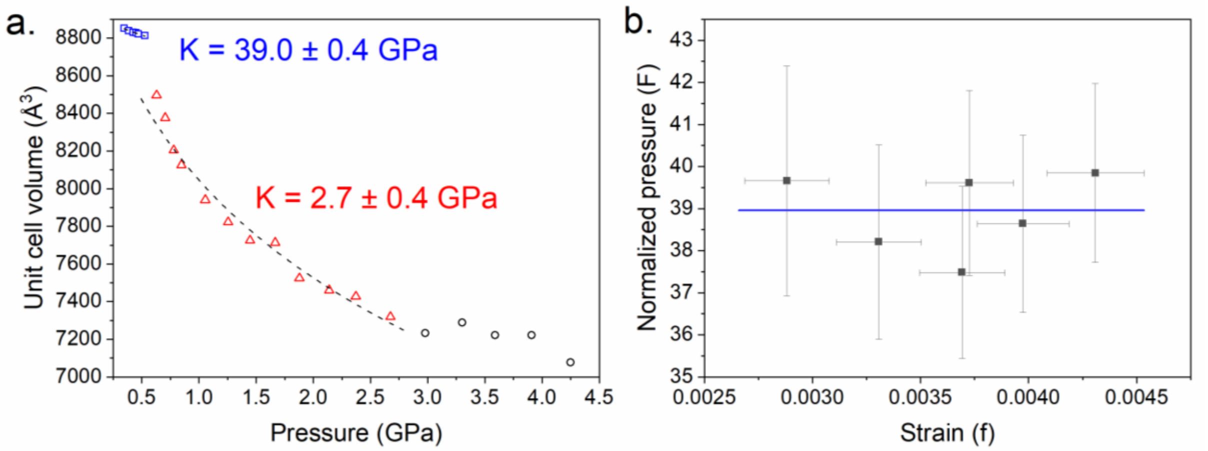

Figure S18. (a) *Birch-Murnaghan equation of state fitting for UiO-66 in the 0-0.55 GPa region (6 blue squares, 2nd order) and 0.55-2.75 GPa region (12 red triangles, 3rd order). Experimentally determined bulk moduli are reported for the corresponding region. Data excluded from fitting are shown by black circles. Error bars are so small as to be obscured by the data points. (b) Ff plot for the 0-0.55 GPa region*

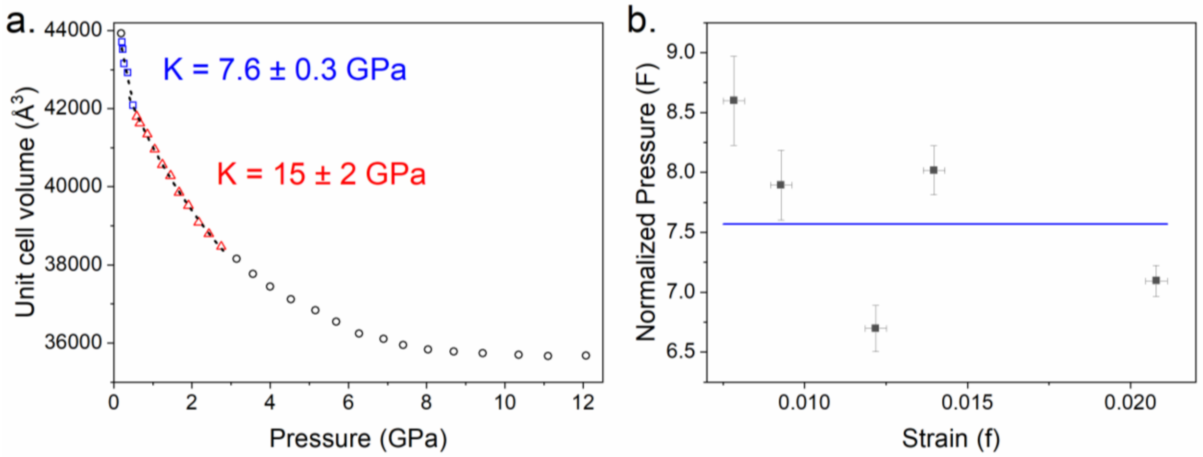

Figure S19. *(a) Birch-Murnaghan equation of state fitting for MOF-808 in the 0-0.55 GPa region (5 blue squares, 2nd order) and 0.55-2.75 GPa region (11 red triangles, 3rd order). Experimentally determined bulk modulus is reported for the corresponding region. Data excluded from fitting are shown by black circles. Error bars are so small as to be obscured by the data points. (b) Ff plot for the 0-0.55 GPa region*

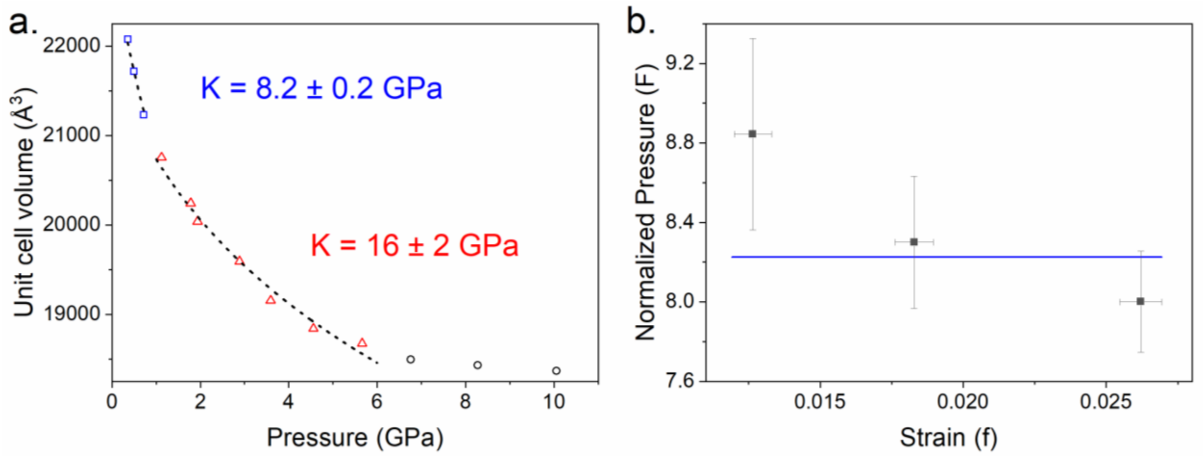

Figure S20. *(a) Birch-Murnaghan equation of state fitting for NU-1000 in the 0-0.75 GPa region (3 blue squares, 2nd order) and 0.75-6 GPa region (7 red triangles, 3rd order). Experimentally determined bulk modulus is reported for the corresponding region. Data excluded from fitting are shown by black circles. Error bars are so small as to be obscured by the data points. (b) Ff plot for the 0-0.55 GPa region*

References

(1) Wang, T. C.; Vermeulen, N. A.; Kim, I. S.; Martinson, A. B. F.; Fraser Stobbart, J.; Hupp, J. T.; Farha, O. K. Scalable synthesis and post-modification of a mesoporous metal-organic framework called NU-1000. *Nature Protocols* **2016**, *11*, 149-162.

(2) Islamoglu, T.; Otake, K. I.; Li, P.; Buru, C. T.; Peters, A. W.; Akpinar, I.; Garibay, S. J.; Farha, O. K. Revisiting the structural homogeneity of NU-1000, a Zr-based metal-organic framework. *Cryst. Eng. Comm.* **2018**, *20*, 5913-5918.

(3) Shearer, G. C.; Chavan, S.; Ethiraj, J.; Vitillo, J. G.; Svelle, S.; Olsbye, U.; Lamberti, C.; Bordiga, S.; Lillerud K. P. Tuned to perfection: Ironing out the defects in metal-organic framework UiO-66. *Chemistry of Materials* **2014**, *26*, 4068-4071.

(4) Katz, M. J.; Brown, Z. J.; Colón, Y. J.; Siu, P. W.; Scheidt, K. A.; Snurr, R. Q.; Hupp, J. T.; Farha, O. K. A facile synthesis of UiO-66, UiO-67 and their derivatives. *Chem. Commun.* **2013**, *49*, 9449-9451.

(5) Castillo-Blas, C.; Romero-Muñiz, I.; Mavrandonakis, A.; Simonelli, L.; Platero-Prats, A. E. Unravelling the local structure of catalytic Fe-oxo clusters stabilized on the MOF-808 metal organic-framework. *Chem. Commun.* **2020**, *56*, 15615-15618.

(6) Cavka, J. H.; Jakobsen, S.; Olsbye, U.; Giullon, N.; Lamberti, C.; Bordiga, S.; Lillerud, K. P. A new Zirconium inorganic building brick forming metal organic frameworks with exceptional stability. *J. Am. Chem. Soc.* **2008**, *130*, 13850-13851.

(7) Mondloch, J. E.; Bury, W.; Fairen-Jimenez, D.; Kwon, S.; DeMarco, E. J.; Weston, M. H.; Sarjeant, A. A.; Nguyen, S. T.; Stair, P. C.; Snurr, R. Q.; Farha, O. K.; Hupp, J. T. Vapor-phase metalation by atomic layer deposition in a metal-organic framework. *J. Am. Chem. Soc.* **2013**, *135*, 10294-10297.

(8) Zheng, H. Q.; Liu, C. Y.; Zeng, X. Y.; Chen, J.; Lü, J.; Lin, R. G.; Cao, R.; Lin, Z. J.; Su, J. W. MOF-808: A Metal-Organic Framework with Intrinsic Peroxidase-Like Catalytic Activity at Neutral PH for Colorimetric Biosensing. *Inorg. Chem.* **2018**, *57*, 9096–9104.

(9) Bristow, J. K.; Svane, K. L.; Skelton, J. M.; Gale, J. D., Walsh, A. Free Energy of Ligand Removal in Metal-Organic Framework UiO-66. *Journal of Physical Chemistry C* **2016**, *120*, 9276-9281.

(10) Shearer, G. C.; Forselv, S.; Chavan, S.; Bordiga, S.; Mathisen, K.; Bjørgen, M.; Svelle, S.; Lillerud, K. P. In Situ Infrared Spectroscopic and Gravimetric Characterisation of the Solvent Removal and Dehydroxylation of the Metal Organic Frameworks UiO-66 and UiO-67. *Topics in Catalysis* **2013**, *56*, 770–782.

(11) Hu, Z.; Kundu, T.; Wang, Y.; Sun, Y.; Zeng, K.; Zhao, D. Modulated Hydrothermal Synthesis of Highly Stable MOF-808(Hf) for Methane Storage. *ACS Sustain. Chem. Eng.* **2020**, *8*, 17042–17053.

(12) Lázaro, I. A. A Comprehensive Thermogravimetric Analysis Multifaceted Method for the Exact Determination of the Composition of Multifunctional Metal-Organic Framework Materials. *Eur. J. Inorg. Chem.* **2020**, *2020*, 4284–4294.

(13) Su, Z.; Miao, Y. R.; Zhang, G.; Miller, J. T.; Suslick, K. S. Bond Breakage under Pressure in a Metal Organic Framework. *Chem. Sci.* **2017**, *8*, 8004–8011.

(14) Zeleňák, V.; Vargová, Z.; Györyová, K. Correlation of Infrared Spectra of Zinc(II) Carboxylates with Their Structures. *Spectrochim Acta A: Mol. Biomol. Spectrosc.* **2007**, *66*, 262–272.

(15) Dewaele, A.; Loubeyre, P.; Mezouar, M. Equations of State of Six Metals above 94 GPa. *Phys. Rev. B: Condens. Matter. Mater. Phys.* **2004**, *70*, 094112.

(16) Otake, K.; Cui, Y.; Buru, C. T.; Li, Z.; Hupp, J. T.; Farha, O. K. Single-Atom-Based Vanadium Oxide Catalysts Supported on Metal–Organic Frameworks: Selective Alcohol Oxidation and Structure–Activity Relationship. *J. Am. Chem. Soc.* **2018**, *140*, 8652–8656.

(17) Wang, X.; Li, Z.; Chen, C.; Wang, K.; Han, B.; Zhou, Q.; Li, F. High Pressure Raman Spectra of Silicone Oil†. *Chemical Journal of Chinese Universities* **2014**, *35*, 2384.
